# Neurohypophysial and paracrine vasopressinergic signaling regulates aquaporin trafficking to hydrate marine teleost oocytes

**DOI:** 10.3389/fendo.2023.1222724

**Published:** 2023-08-11

**Authors:** Alba Ferré, François Chauvigné, Magdalena Gozdowska, Ewa Kulczykowska, Roderick Nigel Finn, Joan Cerdà

**Affiliations:** ^1^ Institute of Agrifood Research and Technology (IRTA)-Institute of Biotechnology and Biomedicine (IBB), Universitat Autònoma de Barcelona, Barcelona, Spain; ^2^ Institute of Marine Sciences, Spanish National Research Council (CSIC), Barcelona, Spain; ^3^ Department of Genetics and Marine Biotechnology, Institute of Oceanology, Polish Academy of Sciences, Sopot, Poland; ^4^ Department of Biological Sciences, University of Bergen, Bergen, Norway

**Keywords:** aquaporin, trafficking, vasopressin, oxytocin, progestin, oocyte hydration, teleost

## Abstract

The dual aquaporin (Aqp1ab1/Aqp1ab2)-mediated hydration of marine teleost eggs, which occurs during oocyte meiosis resumption (maturation), is considered a key adaptation underpinning their evolutionary success in the oceans. However, the endocrine signals controlling this mechanism are almost unknown. Here, we investigated whether the nonapeptides arginine vasopressin (Avp, formerly vasotocin) and oxytocin (Oxt, formerly isotocin) are involved in marine teleost oocyte hydration using the gilthead seabream (*Sparus aurata*) as a model. We show that concomitant with an increased systemic production of Avp and Oxt, the nonapeptides are also produced and accumulated locally in the ovarian follicles during oocyte maturation and hydration. Functional characterization of representative Avp and Oxt receptor subtypes indicates that Avpr1aa and Oxtrb, expressed in the postvitellogenic oocyte, activate phospholipase C and protein kinase C pathways, while Avpr2aa, which is highly expressed in the oocyte and in the follicular theca and granulosa cells, activates the cAMP-protein kinase A (PKA) cascade. Using *ex vivo, in vitro* and mutagenesis approaches, we determined that Avpr2aa plays a major role in the PKA-mediated phosphorylation of the aquaporin subdomains driving membrane insertion of Aqp1ab2 in the theca and granulosa cells, and of Aqp1ab1 and Aqp1ab2 in the distal and proximal regions of the oocyte microvilli, respectively. The data further indicate that luteinizing hormone, which surges during oocyte maturation, induces the synthesis of Avp in the granulosa cells *via* progestin production and the nuclear progestin receptor. Collectively, our data suggest that both the neurohypophysial and paracrine vasopressinergic systems integrate to differentially regulate the trafficking of the Aqp1ab-type paralogs *via* a common Avp-Avpr2aa-PKA pathway to avoid competitive occupancy of the same plasma membrane space and maximize water influx during oocyte hydration.

## 1 Introduction

Water transport during vertebrate oogenesis determines the phenotypic nature of antral follicles in mammals and the pelagic (floating) eggs of marine teleosts (1, 2). In both cases, aquaporins are recognized as the primary channels of transmembrane water flux resulting in the fluid-driven expansion of the follicles (2, 3). However, little is known of the endocrine signals regulating their follicular trafficking. Aquaporins belong to a large family of integral membrane proteins that evolved to facilitate the flux of water and other small molecules through their central pores (4). They function in tetrameric assemblages, with each aquaporin monomer that forms a water pore comprised of six transmembrane domains, two hemi-helices bearing conserved Asn-Pro-Asp (NPA) motifs, three extracellular loops (A, C, E), two intracellular loops (B, D), and intracellular N- and C-terminal domains of varying lengths (5). Up to 15 subfamilies (AQP0-14) are recognized in egg-laying mammals, but due to gene loss, only 13 subfamilies (AQP0-12) remain in placental mammals (6). Conversely, although a similar number of orthologous subfamilies have been identified in teleost fishes, the gene copy numbers are higher (19-26 in diploids, 38-48 in paleotetraploids) due to extra rounds of whole genome duplications combined with cladal- and lineage-level tandem duplications and gene losses (6–12).

In mammalian follicles AQP1, -5, -7, -8 and -9 are thought to mediate water transport *via* the granulosa cells to form the extracellular antrum (3, 13, 14). In contrast, in marine teleosts water flows intracellularly into the oocyte across the plasma membrane *via* tandemly duplicated orthologs of mammalian AQP1 (Aqp1ab1 and/or Aqp1ab2) (12, 15–18). The Aqp1ab-type paralogs are encoded in an ancient teleost-specific aquaporin-1 cluster (TSA1C: *aqp1aa-aqp1ab2-aqp1ab1*) that evolved ~300 Ma with the emergence of the clade (12). The *aqp1ab*-type genes of the TSA1C are highly selectively retained in marine species that spawn pelagic eggs, and their loop D and C-terminal subdomains co-evolved together with protein kinase (PKA) pathways and 14-3-3ζ-like (YwhazL) binding proteins to establish a two-step regulated trafficking mechanism that avoids competitive occupancy of the same oocyte membrane space (12). This preovulatory process occurs during the maturational break down of the germinal vesicle (GVBD) and meiosis resumption to form highly hydrated pelagic eggs (>90% H_2_O) that are subsequently dispersed in the oceanic currents (12, 19).

With the exception of a study showing that teleost *aqp1ab1* transcription is activated by the nuclear progestin receptor (nPgr) (20), very little is known of the endocrine regulation of water transport in marine teleost oocytes during meiosis resumption. Studies in the freshwater stinging catfish (*Heteropneusteus fossilis*), which spawns partially hydrated benthic eggs (21), have, however, suggested that vasotocinergic mechanisms acting through distinct arginine vasotocin receptors (Avtr1 and Avtr2) could differentially regulate GVBD and ovulation, and aquaporin-mediated oocyte hydration, respectively (22–24). Such receptors are now termed arginine vasopressin receptors (Avpr) that bind vasopressin (formerly vasotocin), which diverged early in vertebrate evolution to form Avpr1, Avpr2 and oxytocin (Oxt, formerly isotocin) receptor (Oxtr) subtypes (25–29). In mammals, the AVPR1 and OXTR subtypes activate phospholipase C (PLC) for calcium signaling and protein kinase (PKC) activation, while the AVPR2 subtype activates the cAMP-PKA-mediated transduction mechanism (30, 31). The orthologous teleost Avpr and Oxtr are more numerous than the four paralogs present in mammals due in part to a specific whole genome duplication at the root of the crown clade (28). Despite only a few studies being conducted to characterize which receptor-mediated pathways are induced, the available evidence indicates that the teleost Avpr1, Avpr2 and Oxtr subtypes regulate the same signal transduction cascades as in mammals (32–35). More recently, two studies using the *Xenopus laevis* oocyte expression system have shown that the Avp-Avpr2aa (formerly Avp2r)-cAMP-PKA axis can regulate the intracellular trafficking of teleost Aqp1ab1 and Aqp14 channels, while Oxt acting through the Oxtrb (formerly Itr) and a PKC-mediated mechanism can regulate the trafficking of Aqp1aa (36, 37).

In the stinging catfish, in addition to the neuroendocrine/reproductive centers of the brain (28), an intraovarian vasopressinergic system has been described that could also be involved in the paracrine regulation of oocyte hydration (22–24, 38). However, although the Aqp1ab2 channel has been implicated in this mechanism (23), no study has yet demonstrated its subcellular localization or indeed that of the Aqp1aa and Aqp1ab1 channels, which are also encoded in the TSA1C of catfishes (12). In contrast to freshwater fishes in which only ~1% produce semi-buoyant eggs, the great majority of modern euacanthomorph marine teleosts, such as the gilthead seabream (*Sparus aurata*), produce highly hydrated pelagic eggs (2, 12, 39), yet the ovarian production of Avp or Oxt has not been reported. Since we have recently observed that PKA- and PKC-mediated signal transduction pathways stimulate relocation of each of the TSA1C channels (Aqp1aa, Aqp1ab1 and Aqp1ab2) in surrogate amphibian oocytes (12), here we investigated the potential roles of the Avp-Avpr and Oxt-Oxtr axes in the regulation of aquaporin trafficking that facilitates oocyte hydration in the seabream.

## 2 Results

### 2.1 A paracrine vasopressinergic and oxytocinergic system is present in the seabream ovary

**Figure 1:**
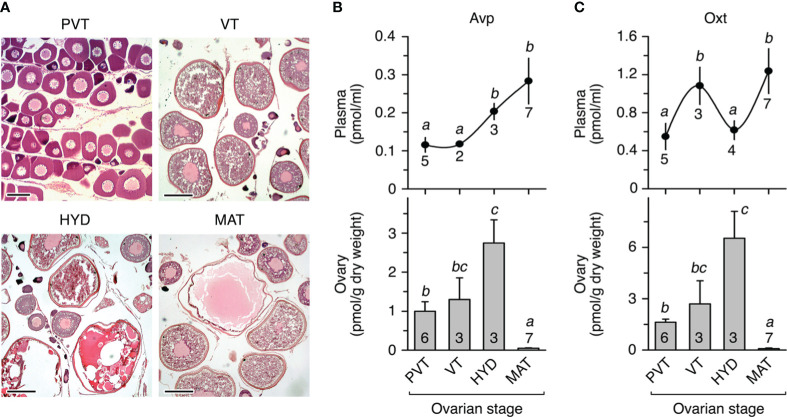
*In vivo* levels of arginine vasopressin (Avp) and oxytocin (Oxt) in seabream plasma and ovary during the natural reproductive cycle. **(A)** Representative photomicrographs of histological sections from ovaries containing ovarian follicle-enclosing oocytes at different developmental stages: previtellogenesis or primary growth stage (PVT), vitellogenesis (VT), oocytes undergoing meiosis resumption and hydration (HYD), and fully matured and hydrated oocytes and ovulated eggs (MAT). Scale bars, 100 µm (PVT), 400 µm (VT-MAT). **(B, C)** Levels of Avp **(A)** and Oxt **(B)** in plasma (upper panels) and ovary (lower panels) from females at the different reproductive stages as shown in **(A)** In each panel, values (mean ± SEM) with different superscript are significantly (ANOVA, *P* < 0.05) different. The number of fish analyzed at each stage are indicated in each plot.

To investigate whether teleost Avp and Oxt are produced locally in the seabream ovary, we initially determined the changes in the concentration of the nonapeptides both in the plasma and in the ovary of females showing different stages of ovarian follicle development (**Figure 1A**) using high performance liquid chromatography (HPLC). Plasma and ovarian levels of Avp progressively increased in females with ovaries at the vitellogenic stage and undergoing meiotic maturation and hydration (**Figure 1B**). However, in females with ovaries containing fully matured and hydrated follicle-enclosed oocytes and ovulated eggs, the ovarian Avp levels markedly dropped, while the plasma levels remained elevated (**Figure 1B**). By contrast, the plasma Oxt levels in females were approximately three times higher than those of Avp and showed a cyclical pattern (**Figure 1C**). Thus, circulating Oxt concentrations were higher during vitellogenesis and the fully mature and hydrated stages compared to other stages (**Figure 1C**). However, the intraovarian changes of Oxt during the reproductive cycle reflected those observed for Avp, with a precipitous drop during the maturation and hydration stage (**Figure 1C**).

**Figure 2:**
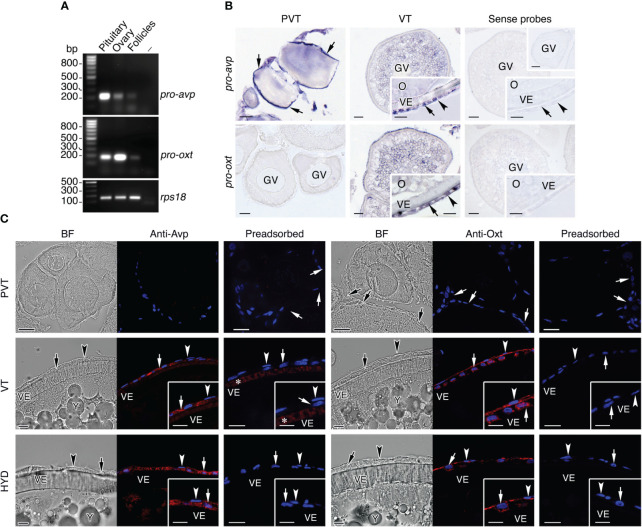
Identification of the cellular sites of Avp and Oxt production in seabream ovarian follicles. **(A)** Representative RT-PCR analysis of the expression of mRNAs encoding the Avp and Oxt pro-peptides (*pro-avp* and *pro-oxt*, respectively) in the seabream ovary and isolated vitellogenic ovarian folliclesand in the pituitary as positive control. The ribosomal protein S18 (*rps18*) was used as the reference. The minus indicates absence of RT during cDNA synthesis. The sizes (bp) of the molecular markers are indicated on the left. **(B)** Localization of *pro-avp* and *pro-oxt* transcripts in previtellogenic (PVT) and vitellogenic (VT) ovarian follicles. Paraffin sections were hybridized with antisense DIG-labeled riboprobes specific for *pro-avp* and *pro-oxt* or sense probes (rigth panels, negative controls). Scale bars: 10 µm (PVT), 20 µm and 8 µm insets (VT). **(C)** Immunostaining of Avp and Oxt peptides (red, right panels) in PVT and VT follicles, and in follicles undergoing meiosis resumption and hydration (HYD). The corresponding brightfield (BF) images are also shown (left panels). The reactions were visualized with Cy3-conjugated sheep anti-rabbit IgG and the nuclei were counterstained with 4’,6-diamidino-2-phenylindole (DAPI; blue). Control sections were incubated with preabsorbed antiserum. In B and C, theca and granulosa cells are indicated by an arrowhead and arrow, respectively. Scale bars, 20 µm (PVT), 10 µm (VT and HYD). Abbreviations: GV, germinal vesicle; O, oocyte; VE, vitelline envelope.

To rule out the possibility that intraovarian Avp and Oxt detected in our analysis were the result of their accumulation from circulating products, we investigated whether these peptides can also be produced within the seabream ovary, as reported for the stinging catfish (38, 40). For this, we first assessed the expression of *pro-avp* and *pro-oxt* genes in ovarian follicles by reverse transcription-polymerase chain reaction (RT-PCR) and *in situ* hybridization (ISH) using gene-specific oligonucleotide primers and riboprobes, respectively. The RT-PCR experiments indicated that *pro-avp* and *pro-oxt* mRNAs were expressed in the pituitary (as a positive control) and the ovary, whereas low but detectable transcripts from both genes were amplified in isolated vitellogenic ovarian follicles (**Figure 2A**). The ISH performed with histological sections of ovaries at the primary growth and vitellogenic stages showed that primordial follicle cells adjacent to previtellogenic oocytes were strongly positive for *pro-avp*, whereas these cells were devoid of *pro-oxt* mRNAs (**Figure 2B**). In vitellogenic ovarian follicles, in which follicle cells are fully differentiated into cuboidal granulosa cells and enlarged and elongated theca cells, *pro-avp* transcripts were detected only in the granulosa cells, while both granulosa and theca cells appear to express *pro-oxt* mRNAs (**Figure 2B**). In the cytoplasm of vitellogenic oocytes, some weak *pro-avp* and *pro-oxt* staining was also detected (**Figure 2B**). For both transcripts, no extrafollicular expression (i.e., interstitial tissue) was noted in the ovarian stages examined, and control sections incubated with sense probes were negative (**Figure 2B**).

The above observations were further confirmed by immunofluorescence microscopy using anti-Avp and anti-Oxt rabbit antisera, which were previously validated by immunostaining of the seabream posterior pituitary gland (**Supplementary Figure S1**). These experiments showed that primordial follicle cells associated to primary growth oocytes were negative for Avp or Oxt immunostaining, and confirmed that granulosa cells surrounding vitellogenic oocytes, as well as those surrounding oocytes undergoing meiotic maturation and hydration, synthesize Avp (**Figure 2C**). In contrast, Oxt immunoreaction was observed in theca and granulosa cells in both follicular stages, in agreement with the ISH data (**Figure 2C**). Preadsorption of the antisera with the corresponding Avp and Oxt peptides led to a complete absence of staining in all the follicular stages analyzed, except a cross-reaction of the Avp antiserum with the vitelline envelope in vitellogenic follicles (**Figure 2C**), indicating the specificity of the signals in the follicular cells.

**Figure 3:**
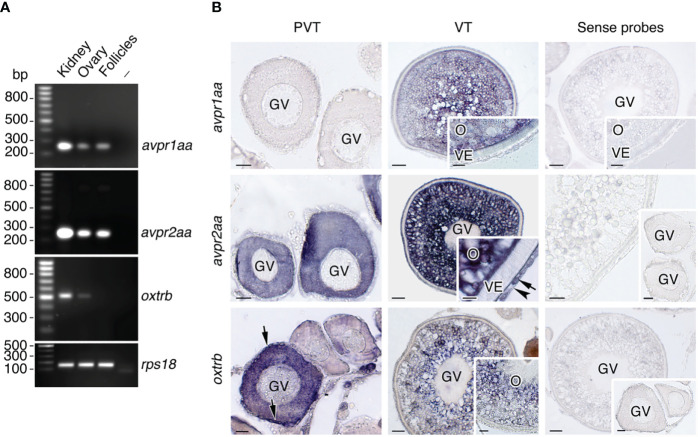
Localization of Avp and Oxt receptor mRNA expression in seabream ovarian follicles. **(A)** Representative RT-PCR analysis of the expression of *avpr1aa*, *avpr2aa* and *oxtrb* mRNAs in the seabream ovary and isolated ovarian follicles, and in kidney as positive control. The ribosomal protein S18 (*rps18*) was used as the reference. The minus indicates absence of RT during cDNA synthesis. The sizes (bp) of the molecular markers are indicated on the left. **(B)** Localization of *avpr1aa*, *avpr2aa* and *oxtrb* transcripts in previtellogenic (PVT) and vitellogenic (VT) ovarian follicles. Paraffin sections were hybridized with specific antisense DIG-labeled riboprobes or sense probes (rigth panels, negative controls). Theca and granulosa cells are indicated by an arrow and arrowhead, respectively. Scale bars, 10 µm (PVT), 20 µm (VT). GV, germinal vesicle; O, oocyte; VE, vitelline envelope.

To assess whether Avprs and Oxtrs are also expressed in ovarian follicles we carried out RT-PCR and ISH using specific primers and riboprobes for two seabream Avpr subtypes, Avpr1aa and Avpr2aa (previously termed Avt1a2r and Avt2r, respectively), and one previously identified Oxtrb (formerly Itr) (41). These receptors were selected as representative Avprs and Oxtrs with a potential for different ligand specificities and the activation of different signal transduction pathways. The results of the RT-PCR assays, using kidney total RNA as a positive control, showed that both *avpr1aa* and *avpr2aa* transcripts could be amplified from the ovary and isolated follicles, while *oxtrb* expression was also noted in the ovary but not detected in follicles (**Figure 3A**). The ISH data indicated that previtellogenic oocytes express *avpr2aa* and *oxtrb*, but not *avpr1aa*, whereas *oxtrb* mRNAs were also observed in the primordial follicle cells (**Figure 3B**). During vitellogenesis, follicles start to express *avpr1aa*, but only in the oocyte and not in the follicle cells, whereas *avpr2aa* expression in oocytes is maintained and activated in the differentiated theca and granulosa cells (**Figure 3B**). At this stage, the *oxtrb* transcripts in oocytes seemed to be slightly reduced and were no longer detected in the follicle cells (**Figure 3B**). For the three transcripts sense probes gave negative results (**Figure 3B**).

Altogether, the above data demonstrate the local production of Avp and Oxt nonapeptides in the seabream ovarian follicle, as well as the expression of their cognate receptors in the follicle cells and/or oocyte, which would be consistent with a role of a vasopressinergic and oxytocinergic regulatory system controlling aquaporin intracellular trafficking and oocyte hydration.

### 2.2 Pharmacological characterization of seabream Avpr1aa, Avpr2aa and Oxtrb receptor subtypes

**Figure 4:**
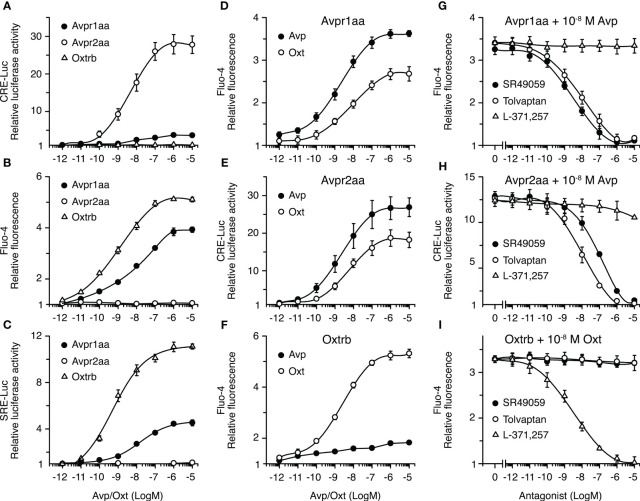
Functional characterization of seabream Avp and Oxt receptors in HEK293T cells. **(A–F)** Activation of the Avpr1aa, Avpr2aa and Oxtrb by graded concentrations of the corresponding ligands **(A–C)**, and ligand selectivity **(D–F)** of each receptor. **(G–I)** Inhibition of the activation of the Avpr1aa, Avpr2aa and Oxtrb by 10^-8^ M of the corresponding ligands in the presence of increasing concentrations of vasopressin (SR49049 and Tolvaptan) and oxytocin (L-371 257) receptor antagonists. In all experiments, hormone-induced cAMP production and PKC activation were indirectly quantified by measuring the luciferase activity from the CRE-Luc and SRE-Luc reporter vectors, respectively, which were cotransfected with the receptor cDNA containing plasmids. Intracellular Ca^2+^ was determined by loading the cells with the Fluo-4 Ca^2+^-sensitive dye before activation of the receptors. The data (mean ± SEM) from each plot are derived from three to six independent transfections.

Since the different Avprs and the Oxtrs are considered to trigger distinct PKA and PKC signaling cascades, and it has been shown that these kinases can regulate seabream Aqp1aa, -1ab1 and -1ab2 trafficking (12, 36), we next investigated the signaling pathways activated by seabream Avpr1aa, Avpr2aa and Oxtrb. For this, human embryonic kidney cells 293T (HEK293T) were transiently transfected with *avpr1aa*, *avpr2aa* or *oxtrb* full-length cDNAs, isolated from the seabream ovary based on publicly available data. Cells were transfected at the same time with β-Galactosidase (β-Gal) together with a cAMP-responsive reporter gene plasmid (pCRE-luc), or a plasmid for activation of the phospholipase C (PLC)/PKC pathway (pSRE-luc), and exposed to increasing doses of the nonapeptides ranging from 10^-12^ to 10^-5^ M (**Figure 4**). The receptor-induced increase in intracellular Ca^2+^ concentration ([Ca^2+^]_i_), as a second messenger in the PLC signal transduction pathway, was determined using Fluo-4 AM, a cell-permeable, fluorescent Ca^2+^ indicator.

In cells expressing the Avpr1aa or Oxtrb receptors, incubation with Avp or Oxt, respectively, resulted in a concentration-dependent increase of pSRE-induced luciferase activity and of [Ca^2+^]_i_, while it did not induce measurable increases in pCRE-mediated luciferase activity (**Figures 4A–C**). However, the Avpr1aa was less affective at activating PKC or increasing the [Ca^2+^]_i_ than the Oxtrb, since the half-maximal effective concentrations (EC_50_) of Avp on its cognate receptor were ~10 times higher compared with those of Oxt on the Oxtrb (**Figures 4B, C**). In contrast, in Avpr2aa expressing cells Avp induced a potent dose-dependent response in pCRE-induced luciferase activity, while PKC was not activated and the [Ca^2+^]_i_ was not affected (**Figures 4A–C**). Receptor cross-activation experiments showed that both Avpr1aa and Avpr2aa could be activated by Avp and Oxt, although the EC_50_ for Oxt on these receptors was ~10 times higher with respect to that of Avp (**Figures 4D, E**). On the contrary, the Oxtrb was more specific for Oxt, since only a slight increase of the [Ca^2+^]_i_ in response to Avp was noted in cells expressing this receptor (**Figure 4F**).

To search for effective and selective inhibitors of seabream Avprs and Oxtrb, which could be used in further physiological experiments, we tested the effect of different non-peptide antagonists selective for mammalian AVPR1A (SR49059) (42), AVPR2 (tolvaptan, TLV) (43) and OXTR (L-371,257) (44). In these experiments, cells were transfected with Avpr1aa, Avpr2aa or Oxtrb and reporter plasmids as above, and preincubated with increasing doses (from 10^-12^ to 10^-5^ M) of the different receptor antagonists, and then subsequently treated with the approximate EC_50_ of Avp or Oxt on each cognate receptor (10^-8^ M). The results showed that activation of the Avpr1aa was equally inhibited by SR49059 and TLV, which showed half maximal inhibitory concentration (IC_50_) of ~10^-9^ M, although the SR49059 was slightly more effective (**Figure 4G**). Both SR49059 and TLV also inhibited the Avpr2aa, although in this case TLV was ~10 times more potent than SR49059 at blocking the receptor (**Figure 4H**). In contrast, the L-371,257 antagonist had no effect on Avpr1aa or Avpr2aa activation (**Figure 4G, H**). Finally, the Oxtrb was well blocked by L-371,257, showing an IC_50_ of ~10^-9^ M, while it was not affected by SR49059 or TLV (**Figure 4I**). These data therefore indicate that TLV and L-371,257 are, respectively, potent and selective inhibitors of seabream Avpr and Oxtr receptor subtypes.

### 2.3 Avp and Oxt differentially regulate the intracellular trafficking of the seabream TSA1C channels

**Figure 5:**
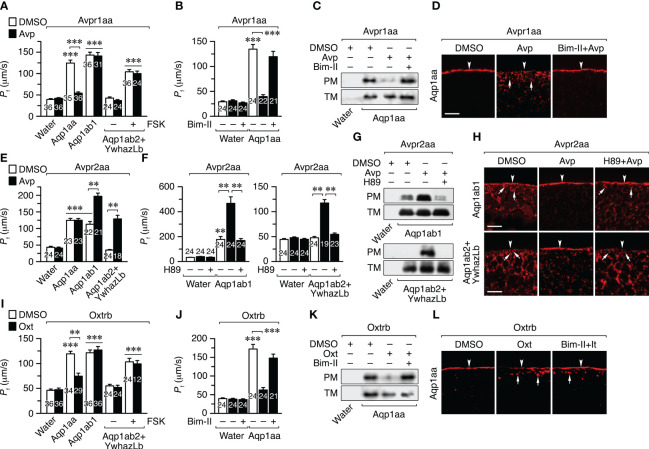
Avp and Oxt regulation of seabream Aqp1aa, Aqp1ab1 and Aqp1ab2 intracellullar trafficking in *X. laevis* oocytes. **(A, B, E, F, I, J)** Osmotic water permeability (*P*_f_) of oocytes expressing the seabream Avpr1aa **(A, B)**, Avpr2aa **(E, F)** or Oxtrb **(I, J)**, and injected with either water, wild-type Aqp1aa, Aqp1ab1 or Aqp1ab2 plus YwhazLb. Oocytes were treated with DMSO (controls) or 10 μM Avp or Oxt **(A, E, I)**, in the presence or absence of 10 μM of PKC and PKA inhibitors (Bim-II and H89, respectively) **(B, F, J)**. Oocytes injected with Aqp1ab2+ YwhazLb were also treated with or without 10 µM forskolin (FSK). Data are the mean ± SEM with the number of oocytes indicated in each bar. **P < 0.01; ***P < 0.001 (unpaired Student’s *t*-test), with respect to water-injected oocytes, or as indicated in brackets. **(C, D, G, H, K, L)** Representative immunoblot **(C, G, K)** of Aqp1aa, Aqp1ab1 and Aqp1ab2 in total (TM) and plasma (PM) membrane extracts from oocyes treated as above, and corresponding immunostaining **(D, H, L)**, using paralog-specific antibodies. The arrowheads indicate the PM, whereas the arrows indicate aquaporin-containing intracellular vesicles. Scale bars, 10 µm.

To investigate whether Avprs and Oxtrb can regulate the intracellular trafficking of Aqp1aa, -1ab1 or -1ab2, we initially employed oocytes from *X. laevis* as a surrogate experimental system (**Figure 5**). Oocytes were injected with *avpr1aa*, *avpr2aa* or *oxtrb* cRNAs, synthesized from human influenza hemagglutinin (HA)-tagged cDNAs, which drives the constitutive expression of the receptors in the oocyte plasma membrane (**Supplementary Figure S2**). To test the effect of receptor activation on the trafficking of the TSA1C channels, each receptor was coinjected with cRNAs encoding Aqp1aa, Aqp1ab1 or Aqp1ab2, or water as a negative control. Oocytes were exposed to Avp or Oxt, depending on the type of receptor expressed, or to the hormone vehicle dimethyl sulfoxide (DMSO) as control, and subsequently submitted to swelling assays to determine the osmotic water permeability (*P*_f_). Since Aqp1ab2 can only traffic to the frog oocyte plasma membrane when it is bound to the teleost-specific YwhazLb protein in the presence of the intracellular cAMP activator forskolin (FSK) (12), Aqp1ab2-injected oocytes were also coinjected with seabream YwhazLb cRNA and, in some experiments, exposed to FSK prior to nonapeptide treatment.

Oocytes expressing the Avpr1aa and Aqp1aa or Aqp1ab1 showed a 3-4-fold increase in *P*_f_ with respect to the control oocytes after a hypoosmotic challenge. However, Avp treatment reduced the *P*_f_ by 80% in oocytes expressing Aqp1aa, but not in those injected with Aqp1ab1 (**Figure 5A**). In the case of oocytes expressing Avpr1aa plus Aqp1ab2, treatment with Avp did not affect the oocyte *P*_f_ triggered by FSK exposure (**Figure 5A**). Preincubation of Avpr1aa+Aqp1aa oocytes with the PKC inhibitor bisindolylmaleimide II (Bim-II) prevented the Avp-induced inhibition of *P*_f_ (**Figure 5B**), in agreement with the activation of the PLC/PKC signaling pathway by the Avpr1aa previously observed in HK293T cells. Immunoblotting of total and plasma membrane extracts of oocytes and immunofluorescence microscopy using Aqp1aa-specific antibodies revealed that the hormone-induced reduction in the oocyte *P*_f_ was due to the internalization of the channel in response to Avpr1aa activation, which was blocked in the presence of Bim-II (**Figures 5C, D**). The same results were obtained in oocytes expressing the Oxtrb and Aqp1aa, Aqp1ab1 or Aqp1ab2 plus YwhazLb (**Figures 5I–L**), indicating that activation of Avpr1aa or Oxtrb by their cognate ligands inhibit Aqp1aa trafficking to the oocyte surface through PKC signaling, while Aqp1ab1 and -1ab2 are not regulated by these receptors.

In contrast, the *P*_f_ of oocytes expressing Avpr2aa and Aqp1ab1 or Aqp1ab2+YwhazLb (in the latter case without FSK pretreatment) was increased by ~2- and ~4-fold, respectively, after Avp exposure, whereas that of the oocytes co-expressing Avpr2aa and Aqp1aa did not change (**Figure 5E**). The Avpr2aa-mediated increase in the *P*_f_ of oocytes expressing Aqp1ab1 or Aqp1ab2+YwhazLb occurred through the Avp-induced insertion of the channels into the oocyte plasma membrane as revealed by immunoblotting and immunolocalization experiments using paralog-specific antibodies (**Figures 5F–H**). However, this trafficking was prevented by the PKA inhibitor H89 (**Figures 5F–H**). These data thus show that Avpr2aa can positively regulate Aqp1ab1 and Aqp1ab2 trafficking to the plasma membrane through the PKA signaling cascade, which is activated in both oocytes and cultured cells.

### 2.4 Molecular basis of the nonapeptide regulation of aquaporin trafficking

**Figure 6:**
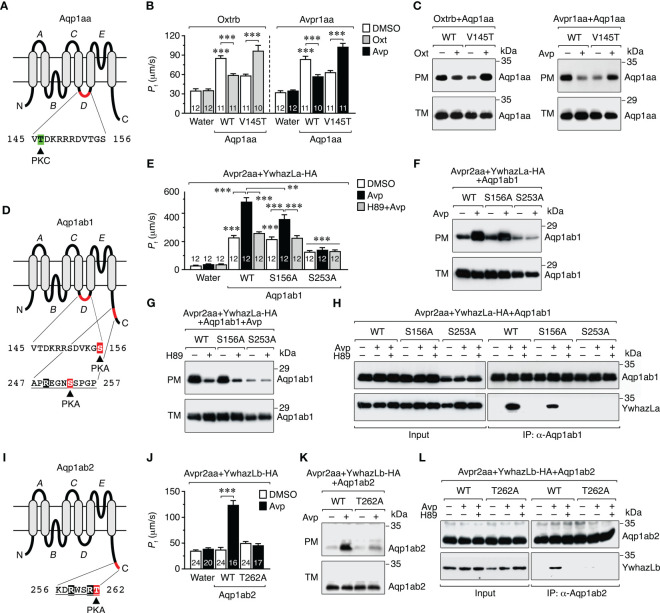
Molecular basis of the vasopressinergic and oxytocinergic regulation of Aqp1aa, Aqp1ab1 and Aqp1ab2 trafficking. **(A, D, I)** Schematic representation of seabream Aqp1aa, Aqp1ab1 and Aqp1ab2 monomers showing the six transmembrane domains, the five connecting loops **(A–E)**, and the PKC and PKA phosphorylation sites involved in channel trafficking (12). The predicted YWHA-binding regions in the Aqp1ab1 and Aqp1ab2 C-termini are underlined. **(B, C)** Osmotic water permeability (*P*_f_) **(B)** and immunoblots of total (TM) and plasma (PM) membrane extracts **(C)** of oocytes expressing the Oxtrb or Avpr1aa and wild-type (WT) Aqp1aa or the Aqp1aa-V145T mutant, and exposed to DMSO (controls) or 10 μM Avp or Oxt. **(E–G)** *P*_f_ of oocytes expressing the Avpr2aa and WT Aqp1ab1 or Aqp1ab1 mutants (S156A and S253A), together with HA-tagged YwhazLa (YwhazLa-HA), and treated with Avp in the presence or absence of 10 µm of H89. The corresponding immunoblots are shown in **(F, G)**. **(H)** Co-immunoprecipitation of WT and mutant Aqp1ab1 channels and YwhazLa-HA in the same oocytes using Aqp1ab1 and HA specific antibodies. **(J, K)** *P*_f_ **(J)** and immunoblot **(K)** of oocytes expressing the Avpr2aa and WT Aqp1ab2 or Aqp1ab2-T262A, together with YwhazLb-HA, and treated with Avp as above. **(L)** Co-immunoprecipitation of both channels in the same oocytes carried out as in **(H)**. Data in B, E and J are the mean ± SEM with the number of oocytes indicated in each bar. **P < 0.01; ***P < 0.001 (unpaired Student’s *t*-test), with respect to water-injected oocytes, or as indicated in brackets. In C, F-H, K and L, molecular mass markers (kDa) are on the right.

The above data are consistent with our recent study using direct pharmacological activation of PKC and PKA in *X. laevis* oocytes, which showed that in euacanthomorph teleosts, such as the seabream, PKC negatively regulates Aqp1aa trafficking to the plasma membrane, while PKA positively regulates this mechanism in Aqp1ab1 and Aqp1ab2 (12). This latter study also identified the kinase phosphorylation sites involved in trafficking regulation of the different channels: Thr^145^ (loop D) in Aqp1aa, Ser^156^ and Ser^253^ (intracellular loop D and C-terminus, respectively) in Aqp1ab1, and Thr^262^ (C-terminus) in Aqp1ab2 (**Figures 6A, D, I**). However, plasma membrane trafficking of Aqp1ab1 partially depends on channel binding to the teleost-specific YwhazLa, through PKA phosphorylation of Ser^253^ within the YWHA binding motif, while that of Aqp1ab2, as mentioned above, strictly relies on PKA-mediated Thr^262^ phosphorylation and the YwhazLb interaction (12) (**Figures 6A, D, I**). Therefore, in order to investigate whether Avp and Oxt control Aqp1aa, Aqp1ab1 and Aqp1ab2 trafficking through the same mechanisms, *X. laevis* oocytes were injected with cRNAs for *avpr1aa*, *avpr2aa* or *oxtrb* and wild-type TSA1C channels or mutant forms in which the PKA and PKC phosphorylation sites were replaced by non-phosphorylatable Ala residues (12), together with HA-tagged YwhazLa or -zLb. The *P*_f_ of oocytes, the trafficking of the channels to the oocyte plasma membrane, and the interaction of Aqp1ab1 and Aqp1ab2 with YwhazLa or -zLb carrier proteins, respectively, in response to the nonapeptides, were then examined by using swelling assays, immunoblotting and co-immunoprecipitation using Aqp1ab1- and Aqp1ab2-specific antibodies.

The results of these experiments showed that the reduction of oocyte permeability and channel accumulation in the plasma membrane in response to Avp or Oxt, in oocytes expressing wild-type Aqp1aa plus Avpr1aa or Oxtrb, was completely reversed when oocytes expressed the Aqp1aa-V145T mutant instead of the wild-type (**Figures 6B, C**), as observed when PKC is directly activated in Aqp1aa expressing oocytes (12). In oocytes expressing wild-type Aqp1ab1, YwhazLa-HA and Avpr2aa, Avp triggered a more robust increment in the oocyte *P*_f_ and of the relative amount of the channel in the plasma membrane (**Figures 6E, F**) than in oocytes without YwhazLa (**Figure 5E**), which was partially or completely prevented, respectively, in oocytes expressing the Aqp1ab1-S156A or -S253A mutants (**Figures 6E, F**). However, the positive effect of Avp on plasma membrane trafficking of both wild-type Aqp1ab1 and Aqp1ab1-S156A channels was equally abolished in the presence of the H89 inhibitor (**Figures 6E, G**). Co-immunoprecipitation experiments using the Aqp1ab1 antibody confirmed that Avp promoted the interaction of wild-type Aqp1ab1 and Aqp1ab1-S156A with YwhazLa, which was inhibited in both cases in the presence of H89 (**Figure 6H**). However, the Aqp1ab1-S253A mutant, which did not traffic to the oocyte surface after Avp stimulation, was not able to interact with YwhazLa regardless of H89 treatment (**Figure 6H**). These data agree with our previous study (12) and suggest that Avp-mediated targeting of Aqp1ab1 to the plasma membrane depends on PKA phosphorylation of C-terminal Ser^253^, which drives YwhazLa binding, and of loop D Ser^156^ once the complex Aqp1ab1-YwhazLa is formed. In contrast, oocytes expressing Aqp1ab2 and Avpr2aa, together with YwhazLb-HA, only showed a higher *P*_f_, together with an increased channel expression in the oocyte plasma membrane and channel binding to YwhazLb, after Avp treatment, which were all blocked in oocytes expressing the Aqp1ab2-T262A mutant (**Figures 6J–L**). These findings therefore indicate that the Avp-mediated regulation of Aqp1ab2 trafficking is specifically mediated by PKA phosphorylation of Thr^262^ in the C-terminus, allowing YwhazLb binding and further insertion of the channel into the plasma membrane.

### 2.5 Intraovarian Avp production is regulated by gonadotropin-induced progestin

**Figure 7:**
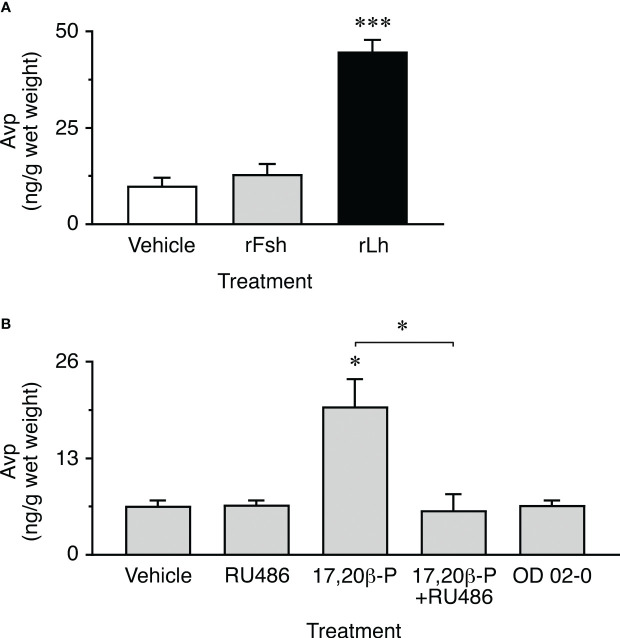
Gonadotropin and progestin induction of ovarian Avp production *in vitro*. **(A)** Amounts of Avp measured in ovarian extracts after ~20 h of exposure to 100 ng/ml of single-chain recombinant seabass Fsh or Lh (rFsh and rLh, respectively), or to the hormone vehicle (controls). Data are the mean ± SEM (*n* = 10 replicates compiled from two separated experiments on two females). **(B)** Avp content in ovarian explants incubated with 0.1 µg/ml 17,20β-P, 100 µM of the nuclear progesterone receptor inhibitor RU486, 17,20β-P+RU486, or to 10 µM of the membrane progestin receptor agonist OD 02-0. Controls were treated with 0.5% ethanol. Values are the mean ± SEM (*n* = 4 independent experiments on different females). *P < 0.05; ***P < 0.001 (unpaired Student’s *t*-test), with respect to vehicle exposed explants, or as indicated in brackets.

The previous experiments show that seabream ovarian follicles can synthesize Avp, and that the ovarian levels of the nonapeptide *in vivo* increase during the stage of oocyte maturation and hydration. These observations would be compatible with a role of Avp regulating the intracellular trafficking of Aqp1ab1 and Aqp1ab2 in maturing oocytes, as observed in surrogate amphibian oocytes. However, our data also suggest that the local production of Avp may be under gonadotropic regulation, since a surge of the circulating luteinizing hormone (Lh) typically occurs in seabream females during the maturation stage (45, 46). To test this hypothesis, we carried out *in vitro* experiments in which the production of Avp by postvitellogenic ovarian explants in response to piscine single-chain recombinant Lh (rLh) was evaluated by a custom made Avp enzyme-linked immunosorbent assay (ELISA) (**Supplementary Figure S3**). To investigate the specificity of the rLh potential regulation, in these experiments we also tested the effect of single-chain recombinant follicle-stimulating hormone (rFsh). The results showed that rLh increased the concentration of Avp in the explants by ~4-fold with respect to the water-exposed explants (controls), while the Avp levels in the rFsh-treated explants did not differ from the controls (**Figure 7A**).

Since the progestin 17α,20β-dihydroxypregn-4-en-3-one (17,20β-P) is known to be the maturation-inducing steroid secreted by granulosa cells in response to Lh in sparids (47), we further investigated whether 17,20β-P could mediate the Lh induction of Avp synthesis in postvitellogenic ovarian explants. In addition, to examine the type of progestin receptor potentially involved in this mechanism, treatment with 17,20β-P was applied in the presence or absence of the nuclear progesterone receptor inhibitor RU486 (20), while other explants were treated with the membrane progestin receptor agonist OD 02-0 (10- ethenyl-19-norprogesterone) (48) in the absence of 17,20β-P. The results of these trials confirmed that 17,20β-P induced a 3-fold increase in Avp synthesis in ovarian explants, comparable to that observed in response to rLh, which could be completely abolished by RU486, and not reproduced by OD 02-0 treatment (**Figure 7B**). These data therefore suggest that the specific Lh induction of Avp synthesis in the granulosa cells of seabream postvitellogenic follicles is mediated by a genomic mechanism through the nuclear progestin receptor.

### 2.6 The Avp-Avpr2aa-PKA signaling pathway differentially regulates Aqp1ab1 and Aqp1ab2 trafficking in postvitellogenic seabream oocytes

**Figure 8:**
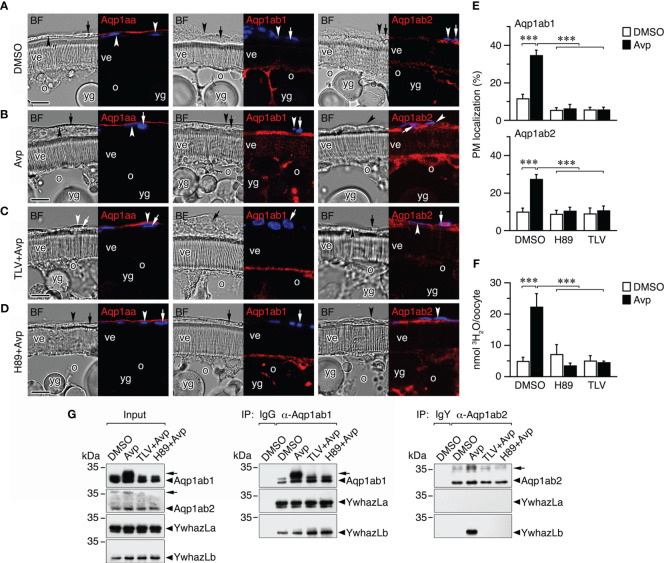
Avp regulation of aquaporin trafficking in seabream ovarian follicles *in vitro*. **(A–D)** Representative brightfield (BF, left panels) and immunostaining of Aqp1aa, Aqp1ab1 and Aqp1ab2 (red color, right panels) in follicles exposed to 10 µM Avp, in the presence or absence of 10 µM of the Avpr1aa and Avpr2aa antagonist Tolvaptan (TLV) or the PKA inhibitor H89. The immunostaining of follicles incubated with TLV or H89 alone are shown in **Supplementary Figure S4**. The nuclei were counterstained with DAPI (blue color). The theca and granulosa cells are indicated by an arrowhead and arrow, respectively. ve, vitelline envelope; o, oocyte; yg, yolk globules. Scale bars, 10 µm. **(E)** Percentage (mean ± SEM; *n* = 5 follicles) of Aqp1ab1 and Aqp1ab2 in the oocyte plasma membrane (PM) after each treatment determined by image analysis. **(F)** Uptake (mean ± SEM; *n* = 3 pools of 10 oocytes) of ^3^H_2_O by follicle-enclosed oocytes treated with Avp as in E. ***P < 0.001 (unpaired Student’s t-test) as indicated in brackets. **(G)** Co-immunoprecipitation of Aqp1ab1 and Aqp1ab2 with YwhazL proteins in ovarian follicles (*n* = 10) treated as in A-D. Left panel shows the immunoblotting of Aqp1ab1, Aqp1ab2, YwhazLa and YwhazLb in the input extracts before immunoprecipitation. The middle and right panels show co-immunoprecipitated YwhazLa and/or YwhazLb with Aqp1ab1 and Aqp1ab2. Controls were immunoprecipitated with IgG or IgY for the Aqp1ab1 and Aqp1ab2 antibodies, respectively. The arrowheads indicate phosphorylated forms of Aqp1ab1 and Aqp1ab2. Molecular mass markers (kDa) are on the left.

To investigate the physiological significance of the Avp regulation of the TSA1C channel trafficking in seabream oocytes, we determined the dynamics of the subcellular localization of endogenous Aqp1aa, Aqp1ab1, and Aqp1ab2, and their interactions with YwhazLa and -zLb proteins, after Avp treatment *in vitro*, using the Aqp1ab1- and Aqp1ab2-specific antisera and commercial mammalian YWHAZ antibodies specific for seabream YwhazLa/b or -zLb (12). For this, we incubated isolated seabream vitellogenic ovarian follicles with Avp, in the presence or absence of the seabream Avpr1aa and Avpr2aa antagonist TLV or the PKA inhibitor H89, while control follicles were exposed to DMSO vehicle alone (**Figure 8**).

The experiments showed that Avp did not induce meiotic maturation or oocyte hydration, in the latter case because the osmotic drive generated by yolk hydrolysis was not activated by the nonapeptide. Immunofluorescence microscopy indicated that Aqp1aa was localized to the epithelium surrounding the ovarian follicles, and not in the theca cell as previously reported (12), and that this localization did not appear to change with Avp treatment (**Figures 8A, B**). In contrast, both Aqp1ab1 and Aqp1ab2 were more accumulated in the most cortical cytoplasmic region of the oocytes, just below the plasma membrane, while Aqp1ab2 was also localized in the granulosa and theca cells (**Figures 8A, B**). After Avp exposure, Aqp1ab1 immunoreactivity appeared more concentrated in the most distal part of the microvilli crossing the vitelline envelope, whereas the Aqp1ab2 channel became localized in a more basal region of the microvilli (**Figures 8A, B**). The differential spatial localization of Aqp1ab1 and Aqp1ab2 in the oocyte microvilli in response to Avp mimics that observed in FSK-treated postvitellogenic follicles (12). However, it was prevented in follicle-enclosed oocytes preincubated with TLV or H89 prior to Avp treatment (**Figures 8C, D**). These observations were corroborated by Aqp1ab1 and Aqp1ab2 fluorescence quantification in the oocyte plasma membrane (**Figure 8E**), as well as by radiolabeled water uptake assays, which showed that water permeability of Avp-treated follicles was higher than that of follicles treated with DMSO alone and reduced in the presence of Avp plus the TLV or H89 inhibitors (**Figure 8F**).

The potential changes in the phosphorylation state of Aqp1ab1 and Aqp1ab2 channels, and of their interactions with YwhazLa and -zLb carrier proteins, during Avp treatment were subsequently investigated by co-immunoprecipitation and immunoblotting. These trials confirmed previous observations (12, 16) indicating that in control postvitellogenic follicles, when Aqp1ab1 is held in the most cortical region of the oocyte and some inserted in the basal region of the microvilli, the channel is already phosphorylated and bound to both YwhazLa and -zLb (**Figure 8G**). However, after Avp treatment Aqp1ab1 becomes more phosphorylated, while maintaining the interaction with YwhazLa and -zLb (**Figure 8G**), which coincides with the secondary loop D Ser^156^ phosphorylation and trafficking of the channel to the most distal region of the oocyte microvilli. Phosphorylation of Aqp1ab2 was also increased after Avp stimulation, but in this case the YwhazLb interaction was only detected in the presence of the nonapeptide (**Figure 8G**), when the channel is translocated to the basal region of the oocyte microvilli. The Avp-mediated increase in the phosphorylation of Aqp1ab1 and Aqp1ab2, as well as the binding of the latter channel to YwhazLb, was blocked by TLV and H89 (**Figure 8G**), suggesting that the differential membrane trafficking regulation of the channels is regulated by the Avpr2aa-PKA transduction pathway.

## 3 Discussion

In this study we show for the first time that in addition to the hypophysial neuroendocrine system, a paracrine vasopressinergic and oxytocinergic signaling system exists in the ovary of a marine teleost. Since expression of Avp and/or Oxt precursor mRNAs or proteins are also expressed in the ovaries of freshwater catfishes (24, 28, 38, 40), as well as in lampreys (25), sharks (49), birds (50), and mammals (51, 52), it seems plausible that an ovarian vasopressinergic and/or oxytocinergic paracrine system existed prior to the emergence of vertebrates.

In the present context, we isolated paralogs from each of the major Avp and Oxt receptor subtypes (Avpr1aa, Avpr2aa and Oxtrb) and functionally characterized the signal transduction pathways they induce in order to determine how the systemic and paracrine systems might integrate to regulate aquaporin-mediated oocyte hydration. The data show that the Avpr1aa and Oxtrb subtypes activate PLC-PKC cascades while the Avpr2aa subtype activates the cAMP-PKA pathway. These results are consistent with the notion that the functional pathway dichotomy between the Avpr1/Oxtr and Avpr2 subtypes may have emerged in the common ancestor of jawed vertebrates (29). However, since teleosts encode up to five copies of the Avpr2 subtype (see **Supplementary Figure S5**), it will be important to validate the pathway specificity of each paralog to avoid expectation bias. For example, in related experiments, our ligand specificity analyses show that seabream Avpr1aa and Avpr2aa display some promiscuity for Avp and Oxt. By contrast, the Avpr1ab ortholog of the white sucker (*Catostomus commersoni*) is only activated by Avp (32). Similarly, our data for the seabream Oxtrb show a high specificity for Oxt, while the white sucker Oxtra can also be activated by Avp, albeit with lower potency (33).

**Figure 9:**
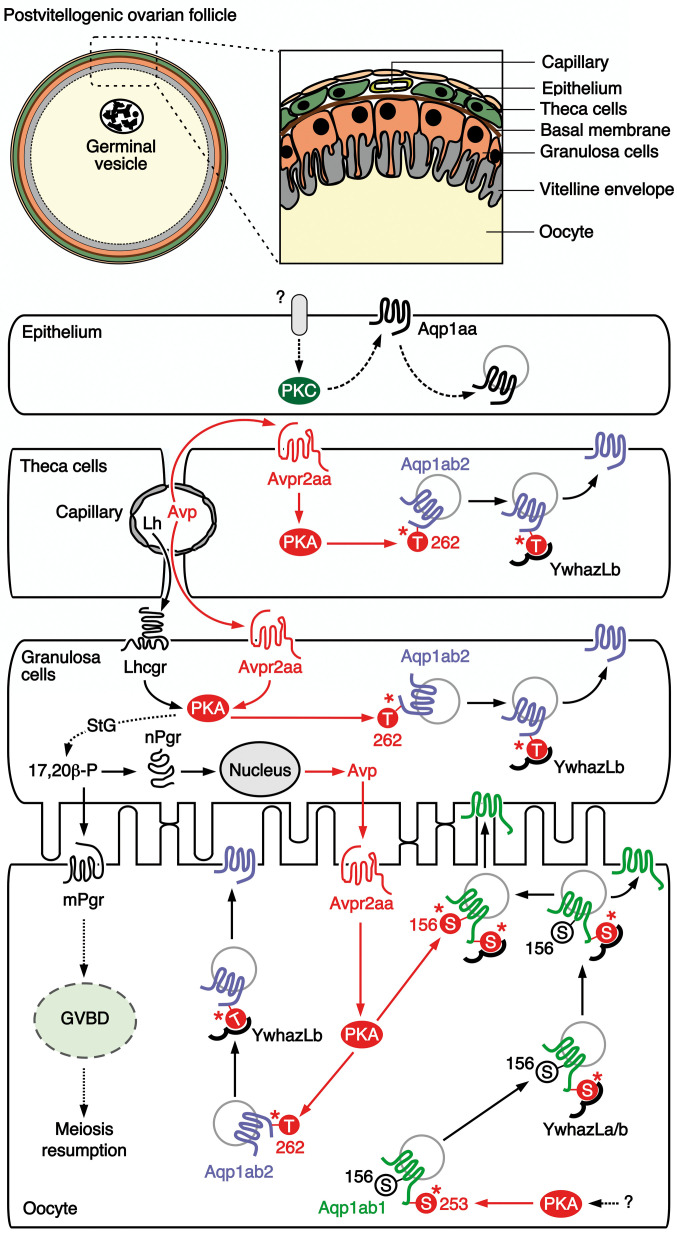
Proposed model for the vasopressinergic regulation of Aqp1ab1 and Aqp1ab2 function in seabream ovarian follicles during meiosis resumption and oocyte hydration. In primary growth oocytes, Aqp1ab1 is phosphorylated at Ser^253^ by protein kinase A (PKA), which is activated by a yet unknown signaling pathway, to bind YwhazLa and -zLb. The complex is then slowly transported to the oocyte cortical region during the vitellogenic period, where it is retained just below the oocyte plasma membrane in postvitellogenic oocytes, while some of Aqp1ab1 enters the basal region of the oocyte microvilli. The release of circulating hypohysial Avp and luteinizing hormone (Lh) from capillaries in the theca layer at the time of oocyte maturation activate the Avpr2aa in theca and granulosa cells, as well as the Lh/choriogonadotropin receptor (Lhcgr) in the granulosa cells. In the theca and granulosa, the Avp-stimulated Avpr2aa triggers PKA activation, which mediates the phosphorylation of Thr^262^ in Aqp1ab2 promoting the binding to YwhazLb and the trafficking of the channel to the plasma membrane of these cells. The activation of the Lhcgr-PKA pathway in granulosa cells drives the synthesis of the progestagen 17,20β-P, through a switch in the steroidogenic (StG) pathway from estrogen to progestagen production. The 17,20β-P, in turn stimulates the local synthesis of Avp through a genomic mechanism mediated by the nuclear progestin receptor (nPgr). The resulting release of Avp and 17,20β-P from the granulosa cells respectively activates the Avpr2aa and the membrane progestin receptor (mPgr) in the oocyte surface. The mPr triggers meiosis resumption (oocyte maturation), whereas the Avpr2aa-PKA signaling regulates the trafficking of Aqp1ab2 as in follicle cells, and a secondary phosphorylation of Ser^156^ in the loop D of Aqp1ab1 channels already complexed with YwhazLa/b. The secondary phosphorylation of Aqp1ab1 further shuttles the channel to the most distal region of the oocyte microvilli. Finally, systemic (circulating) and/or local levels of Avp, or a possible Lh-mediated production of Oxt, may potentially activate the protein kinase C (PKC) signaling pathway in the epithelium, through a yet unidentified receptor, to induce the recycling of Aqp1aa. This model integrates both the systemic and paracrine vasopressinergic regulation of the intracellular trafficking of Aqp1ab-type paralogs in the seabream oocyte, such that competitive plasma membrane spatial occupancy by both channels is avoided and bulk water influx is augmented during oocyte hydration.

Since we had previously established that TSA1C channel trafficking is differentially regulated by the PKC-mediated phosphorylation of loop D sites in Aqp1aa and the PKA-mediated phosphorylation of loop D and C-terminal sites in Aqp1ab1 and the C-terminus of Aqp1ab2 (12), we confirmed here that the same sites control the recycling of Aqp1aa and membrane insertion of the Aqp1ab-type channels when co-expressed with the Avpr1aa/Oxtrb and Avpr2aa subtypes, respectively. By further identifying the pituitary and follicular cell sources of Avp and Oxt together with the ovarian loci of their respective receptors, and monitoring the plasma and ovarian levels of each nonapeptide during oocyte growth and maturation, we can propose a model that is congruent with the suggestions for catfish (24, 28), to explain the neuroendocrine and paracrine regulation of aquaporin trafficking facilitating marine teleost oocyte hydration (**Figure 9**).

In this model, neurohypophysial secretion of Avp throughout oocyte growth (vitellogenesis) arrives at the capillary beds of the thecal layer. From there it diffuses to activate the Avpr2aa receptor, which is expressed in the plasma membrane of the theca and granulosa cells, to induce the PKA-mediated phosphorylation of Thr^262^ in the C-terminus of Aqp1ab2. This results in the coupling of the YwhazLb binding protein and the trafficking of the channel to the cell surface of the theca and granulosa cells. Meanwhile, in the oocyte Aqp1ab1, which is already expressed since the primary growth stage (20) and phosphorylated at the C-terminal Ser^253^ by a PKA-mediated mechanism (12), preferentially couples with the YwhazLa binding protein and is trafficked close to the basal region of the oocyte microvilli during vitellogenesis. Some of Aqp1ab1 also enters the basal region of the oocyte microvilli (12), potentially to mediate water homeostasis during the vitellogenic growth phase. Concomitant with the preovulatory surge of Lh (45, 46), our data show that the circulating and ovarian levels of Avp are increased during meiotic maturation and hydration of the oocytes. Such an elevated systemic Avp further enhances the PKA-mediated trafficking of Aqp1ab2 channels in the theca and granulosa cells. However, as in the catfish (53), the combined activation of Lh on Lhcgr and Avp on Avpr2aa could also multiply the action of the PKA-mediated Lh induction of the steroidogenic shift from estrogen production to the synthesis of the maturation-inducing progestin 17,20ß-P in granulosa cells (54). Thus, the 17,20ß-P could act as a master switch regulating the reciprocal genomic production of Avp *via* the nuclear progestin receptor in granulosa cells, and the non-genomic activation of GVBD and meiosis resumption *via* the membrane progestin receptor that is expressed in the oocyte plasma membrane (55). It is the former reciprocal genomic production of Avp that would provide the paracrine signal between the granulosa cells and the Avpr2aa in the oocyte plasma membrane, which now activates the PKA-mediated phosphorylation of Thr^262^ in the Aqp1ab2 C-terminus and Ser^156^ in loop D of Aqp1ab1. The Thr^262^ phosphorylation promotes the coupling of the YwhazLb binding protein and the trafficking of Aqp1ab2 to the basal region of the oocyte microvilli, while the secondary phosphorylation of loop D Ser^156^ in Aqp1ab1 relocalizes the channel to the distal portion of the oocyte microvilli. In this way, the two Aqp1ab-type paralogs are co-regulated by the same Avp-Avpr2aa-PKA pathway to cooperatively enhance water influx while avoiding competitive occupancy of the same membrane space.

Our proposed model does not yet explain the signal transduction mechanism that may activate PKC-mediated recycling of Aqp1aa in the follicular epithelium, which occurs when this channel is co-expressed with Avpr1aa or Oxtrb in *X. laevis* oocytes. Nevertheless, we did observe that in vitellogenic ovarian follicles, both the mRNA and protein of Oxt are accumulated in the theca and granulosa cells, while those of Avp are only accumulated in the granulosa cells. These observations may suggest that trafficking of epithelial Aqp1aa could also be under Avp or Oxt regulation through receptors not characterized here, such as the Avpr1ab or the Oxtra, since in our study neither *avpr1aa* nor *oxtrb* mRNA expression could not be detected in the follicular epithelial layer. In any event, the potential recycling of the Aqp1aa channel in the follicular epithelium may provide an important mechanism for the flow of water into the hydrating oocyte. The water path originates with the seawater imbibed by the mother and is delivered to the follicles through the thecal capillary beds. By simultaneously recycling epithelial Aqp1aa and driving Aqp1ab2 to the membranes of the theca and granulosa cells, water is only free to flow in one direction toward the oocyte. The differential proteolysis of primarily vitellogenin-Aa (VtgAa) type yolk proteins (39, 56, 57) generates the osmotic driving force that converts the oocyte into the water sink, which then enters *via* the vasopressinergic induction of the Aqp1ab1 and Aqp1ab2 channels to the distal and proximal regions of the microvilli. The water finally becomes trapped within the highly hydrated oocyte due to the precipitous drop in the ovarian levels of Avp, thus ending the paracrine regulation of the trafficking of the Aqp1ab-type channels, and their resultant internalization in the oocyte (12). The subsequent cyclical increase in the level of circulating Oxt may thus prepare the oocytes for ovulation as in mammals (58), leaving the eggs ready for fertilization and their future pelagic passage in the oceanic currents.

In conclusion, the present study uncovered a paracrine vasopressinergic and oxytocinergic system in the ovary of a modern euacanthomorph marine teleost, the gilthead seabream, which supports the notion that this mechanism may have existed prior to the emergence of vertebrates. In both the seabream and catfish, the ovarian vasopressinergic system is possibly activated *via* systemic neuroendocrine signaling to multiply the Lh induction of progestin which generates Avp for the paracrine signaling. In the seabream, however, by functionally characterizing the signal transduction pathways induced by each of the Avp and Oxt receptor subtypes and assaying their ligand specificities, it was possible to uncover the molecular basis of the nonapeptide regulated trafficking of the TSA1C channels. In this respect, the Avpr2aa paralog plays a vital role in the transduction of the paracrine signals and the co-ordinated trafficking of the Aqp1ab-type channels so that they avoid competitive occupancy of the same membrane space and maximize the hydration of the oocyte. It remains to be seen if a similar neuroendocrine/paracrine regulation of aquaporin trafficking exists in older lineages of teleost that retain the Aqp1ab-type paralogs.

## 4 Materials and methods

### 4.1 Experimental animals and sampling

Adult gilthead seabream were obtained from the Institut de Recerca i Tecnologia Agroalimentàries (IRTA) aquaculture facilities (Tarragona, Spain) and transported to the facilities in Institut de Ciències del Mar (Barcelona, Spain). Fish were maintained under natural conditions of photoperiod and temperature and fed three times a week with dry pellets (Seabream Alterna AE, Skretting) and squid *ad libitum*. Samples of blood and gonads were collected throughout the year from sacrificed females as previously described (20), frozen in liquid nitrogen and stored at -80°C. The stage of ovarian development in each female was determined by standard histological analysis as described previously (59).

Adult *X. laevis* were purchased from the Centre de Ressources Biologiques Xénopes (University of Rennes, France) and maintained at the AQUAB facilities of the Universitat Autònoma de Barcelona (UAB, Spain). Frogs were kept in tanks with filtrated freshwater at 18°C, under a 12-h light-dark cycle, and fed two days a week with beef heart or pellets (*Xenopus* Sticks, AQUA Schwarz GmbH, Göttingen, Germany). Oocytes were collected by surgical laparotomy from anesthetized females.

### 4.2 Ligand and receptor nomenclature

The nomenclature used for the seabream vasopressin and oxytocin nonapeptides and corresponding cognate receptors is adopted according to the Human Genome Nomenclature Committee (HGNC) (www.genenames.org), the zebrafish information network (ZFIN) (www.zfin.org) and Ensembl (v109) (www.ensembl.org), according to that previously described (28). The specific nomenclature of the gilthead seabream Avprs and Oxtrs was established through Bayesian inference of two million MCMC generations (aamodel = mixed; burnin = 25%) of a ClustalX amino acid alignment of the seabream Avprs and Oxtrs in relation to 88 actinopterygian orthologs downloaded from Ensembl (v109) (**Supplementary Figure S5**). The resultant tree topology matches that computed by maximum likelihood by Daza et al. (27) with the exception that the zebrafish Avpr2ba and Avpr2bb receptors are named in accordance with the Ensembl (v109) nomenclature.

### 4.3 Antibodies and reagents

Affinity-purified rabbit or chicken polyclonal antibodies specific for seabream Aqp1aa, -1ab1 and -1ab2 have been described elsewhere (12, 60). Antisera against Avp and Oxt were generously provided by Prof. Olivier Kah (INSERM-Université de Rennes 1, France). The HA tag rabbit antibody was from Thermo Fisher Scientific (Invitrogen, #PA1-985), whereas anti-YWHAZ antibodies were purchased from GeneTex (#GTX101075) and Signalway Antibody LLC (#SAB40525), which specificity for seabream YwhazLa and/or YwhazLb has been validated elsewhere (12). Synthetic Avp (CYIQNCPRG-NH_2_) and Oxt (CYISNCPIG-NH_2_) were obtained from PeptideSynthetics (Peptide Protein Research Ltd.). All other reagents were purchased from Merck unless indicated otherwise.

### 4.4 HPLC determination of Avp and Oxt content in plasma and ovary

Ovarian and plasma levels of Avp and Oxt *in vivo* were determined using HPLC with fluorescence detection preceded by solid-phase extraction (SPE). Pieces of ovary (150-350 mg) previously frozen and stored at -80°C were sonicated in 1 ml of Milli-Q water, acidified with 3 μl of glacial acetic acid and incubated in a boiling water bath during 3.5 min. The samples were centrifuged (30 min, 4°C, 10,000 × *g*). Plasma samples (1.5-2 ml each) were acidified with 1 M HCl (100 μl) and centrifuged at 6000·× *g* for 20 min at 4 °C. In both cases, the supernatants were loaded onto previously equilibrated SPE columns (Strata-X, 30 mg/ml; Phenomenex, Torrance, USA) and eluted in 80% acetonitrile. Eluates were evaporated to dryness using TurboVap LVTM (Caliper Life Sciences, PerkinElmer, Inc.), and frozen and stored at -20°C until HPLC analysis. Before quantitative analysis, the dried samples were reconstituted with 0.1 ml of 4% acetic acid and divided into two aliquots for duplicate analysis. For plasma, samples were redissolved in 0.1 ml of acetonitrile:H_2_O (2:1). For pre-column derivatization, 50 μl of sample and 50 μl of 0.05 M borate buffer (pH 8) were mixed, and then 3 μl of NBD-F (4-fluoro-7-nitro-2,1,3-benzoxadiazole; 30 mg/ml of acetonitrile) was added. The solution was heated at 40˚C for 10 min, cooled on ice, and acidified with 5 μl of 1 M HCl. Chromatographic analysis was achieved using Agilent 1200 Series Quaternary HPLC System (Agilent Technology, USA). Peptide separations were performed on a Kinetex C18 column (4.6 x 150 mm, 5 μm, Phenomenex, USA). A gradient elution system was applied for separation of derivatized peptides. The mobile phase consisted of solvent A (0.1% TFA in H_2_O) and solvent B (0.1% TFA in 3:1 acetonitrile:H_2_O). A linear gradient was 40-65% of eluent B in 20 min. Flow rate was set at 1 ml/min and the column temperature was set to 20˚C. Fluorescence detection was carried out at 530 nm with excitation at 470 nm.

### 4.5 Expression constructs

Full-length cDNAs encoding seabream Aqp1aa, Aqp1ab1, Aqp1ab2, YwhazLa and -zLb have been previously isolated (GenBank accession numbers AY626939, AY626938, MW960021, OK572451 and OK572452, respectively). Mutated Aqp1aa, Aqp1ab1 and Aqp1ab2 constructs in PKA and PKC phosphorylation sites were previously produced (12). Full-length cDNAs encoding seabream Avpr1aa, Avpr2aa and Oxtrb were isolated by RT-PCR using intestine or rectum total RNA as previously described (18) and oligonucleotide primers designed based on publicly available sequences (GenBank accession numbers KC195974, KC960488 and KC195973, respectively; **Supplementary Table S1**). The cDNAs were subcloned into the pT7Ts (61) or pcDNA3 (Invitrogen) expression vectors, and fused with an HA epitope tag (YPYDVPDYA) in the C-terminus before the stop codon by using PCR. The final clones were Sanger sequenced to confirm that the constructs were correct.

### 4.6 Pharmacological Avp and Oxt receptor characterization

Seabream Avp and Oxt receptor cDNA constructs in pcDNA3 were transiently expressed in HEK293T cells (ATCC # CRL-11268). The cells were grown in 24 wells plates in DMEM supplemented with penicillin/streptomycin, 2 mM glutamine, and 10% fetal bovine serum (FBS) (Life Technologies) and were incubated at 37°C in 5% CO_2_. Receptor activation was measured using luciferase reporter gene assays and intracellular Ca^2+^ levels. At approximately 50-60% confluence, cells were transfected using Lipofectamine 3000 (Invitrogen) with empty pcDNA3 or Avpr1aa (5 ng), Avpr2aa (5 ng), or Oxtrb (50 ng) cDNAs, 50 ng of pCRE-luc (Agilent Technologies) or 200 ng of pSRE-luc (BD Biosciences Clontech), and 5 ng or 50 ng of β-Gal plasmid (Promega Corp.) to normalize the transfection efficiency. The transfected cells were grown for 36 h at 37°C in an air/CO_2_ [95:5 (v/v)] atmosphere before starvation for 16 h by replacing the DMEM by serum-free DMEM medium. The cells were then incubated in triplicate with different concentrations of Avp or Oxt (from 10^-5^ M to 10^-12^ M), or sterile dimethyl sulfoxide (DMSO) vehicle (0.01% final concentration), for 3 h. In some experiments, the cells were incubated with increasing concentrations (from 10^-12^ to 10^-5^ M) of different AVP (SR49059, Merck #S5701; TLV, Merck #T7455) and OXT (L-371,257, Tocris #2410) receptor antagonists for 1 h prior to stimulation with 10^-8^ M Avp or Oxt. After incubation, the cells were harvested in lysis buffer (25 mM Tris-phosphate, pH 7.8, 2 mM DTT, 10% glycerol, 1% triton X-100), frozen at -80°C for 16-24 h and centrifuged at 14,000 × *g* for 1 min. To determine the luciferase activity, 20 µl of the supernatant was mixed with 100 µl of reconstituted Luciferin (BioThema) in a 96-well plate (Nunc F96 MicroWell Black and White Polystyrene Plate, Thermo Fisher Scientific), and the luminescence measured in an Orion II microplate luminometer (Titertek-Berthold). Luciferase activity was normalized to β-Gal activity measured by colorimetric detection using an Infinite M200 microplate reader (Tecan Group Ltd.). The data was finally expressed as fold-change of luciferase activity with respect to control cells exposed to DMSO only.

To determine the intracellular Ca^2+^ levels after receptor activation we used the cell permeant Fluo-4-AM vital dye (Life technologies #F14201). For this, HEK293T cells were transfected as above with 2.5 µg of Avpr1aa, Avpr2aa or Oxtrb cDNAs in 6-well plates (Thermo Scientific). After 16 h, the cells were transferred to a black 96-well microplate and incubated 24 h at 37°C in an air/CO_2_ [95:5 (v/v)] atmosphere. Fluo-4 AM was diluted in Hank’s Balanced Salt Solution (HBSS) supplemented with glucose, containing 8% NP-40 and 20% DMSO, and added to the cells at 5 µM final concentration. Cells were incubated for 1 h at 37°C in an air/CO_2_ [95:5 (v/v)] atmosphere, and subsequently exposed in triplicate to the nonapeptides, in the presence of absence of inhibitors, as described above. After washing twice with fresh DMEM, fluorescence intensity was measured at excitation and emission wavelengths of 494 and 506 nm, respectively, using an Infinite M200 microplate reader (Tecan Group Ltd.). The background signal of cells incubated with HBSS without Fluo-4 AM was subtracted from each value.

### 4.7 Gene expression analyses

For RT-PCR, total RNA was extracted from different adult tissues, including pituitary, kidney, ovary and isolated ovarian follicles, using the RNeasy Minikit (Qiagen) and DNAse I treatment, following the manufacturer’s instructions. Total RNA (5 μg) was reverse transcribed using 0.5 μg oligo(dT)_17_ primer, 1 mM deoxynucleotide triphosphates (dNTPs), 40 IU RNAse out (Life Technologies Corp.), and 10 IU SuperScript II Reverse Transcriptase enzyme (Life Technologies Corp.) for 1.5 h at 42°C. The PCR was carried out with 1 μl of the RT reaction in a final volume of 50 μl containing PCR buffer with Mg^2+^, 0.2 mM dNTPs, 1 IU of Taq polymerase (Roche), and 0.2 μM of forward and reverse oligonucleotide primers specific for seabream *pro-avp*, *pro-oxt*, *avpr1aa*, *avpr2aa* and *oxtrb* (**Supplementary Table S1**). Reactions were amplified using one cycle at 95°C for 2 min; 35 cycles at 95°C for 30 sec, 60-62°C (depending on primer Tm) for 30 sec, and 1 min at 72°C; and 7 min for final elongation at 72°C. The 18S ribosomal RNA (*rps18*) was used as reference gene. The PCR products were run on 1% agarose gels and photographed.

Cellular localization of *pro-avp*, *pro-oxt*, *avpr1aa*, *avpr2aa* and *oxtrb* expression in ovarian follicles was carried out by ISH using digoxigenin-labelled gene-specific riboprobes as described previously (62). The sense and antisense riboprobes were synthesized using T7- or SP6-RNA polymerases from a PCR-amplified template using specific oligonucleotide primers (**Supplementary Table S1**). The *pro-avp* and *pro-oxt* probes corresponded to the C-terminal end and part of the 3´UTR, or the proximal 3´UTR, respectively. For the receptors, the probes spanned ~300 bp from the middle of the *avpr2aa* cDNA sequence, and ~200 bp of the *avpr1aa* and *oxtrb* cDNAs including the C-terminus and a portion of the 3´UTR.

### 4.8 Functional expression in *X. laevis* oocytes and swelling assays

The cRNAs corresponding to the different constructs were synthesized *in vitro* with T7 RNA polymerase from *Xba*I-digested pT7Ts vector containing the cDNAs. The isolation and microinjection of oocytes, and subsequent swelling assays, were carried out as previously described (63). Oocytes were injected with 50 nl of water alone (controls) or containing 15 ng of Avpr1aa, Avpr2aa or Oxtrb cRNAs. Oocytes expressing the receptors were also injected with wild-type or mutant Aqp1aa (0.1 ng), Aqp1ab1 (0.5 ng) or Aqp1ab2 (25 ng) plus YwhazLb (25 ng). In some experiments, oocytes expressing Avpr2aa and Aqp1ab1 constructs were also injected with YwhazLa (25 ng). The oocyte *P*_f_ was determined after 3 h incubation with 10 µM Avp or Oxt (depending on the type of receptor expressed in oocytes). The role of PKA or PKC on the oocyte *P*_f_ was tested by preincubating the oocytes with 10 µM of H89 or Bim-II for 1 h prior to the addition of the hormones. Oocytes expressing Avpr1aa or Oxtrb together with Aqp1ab2 plus YwhazLb were treated with 10 µM FSK as previously described (12). Control oocytes were treated with 0.1% DMSO.

### 4.9 *In vitro* incubation of ovarian explants and isolated ovarian follicles

Pieces of the ovary at the vitellogenic stage (400-500 mg) were dissected from naturally spawning seabream females and placed in Petri dishes containing 75% Leivovitz L-15 culture medium with L-glutamine and 100 µg/ml gentamicin at pH 7.5. The explants were treated with 100 ng/ml of sea bass (*Dicentrarchus labrax*) rFsh or rLh (64), which activate the seabream Fsh and Lh receptors, respectively (59), 0.1 µg/ml 17,20βP, in the presence or absence of 100 µM of RU486 (Merck M8046), or with 10 µM of OD 02-0 (Axon Medchem, 13258-85-0), for ~20 h at 18°C in a temperature-controlled incubator. Control groups were treated with an equivalent volume of the cell culture medium used for rLh and rFsh production, or with 0.5% ethanol. After incubation, explants were frozen and stored at -80°C until Avp extraction and determination by ELISA.

In other experiments, groups of fully-grown postvitellogenic ovarian follicles (*n* = 10), manually isolated from the ovary, were incubated with 10 µM Avp in the same culture medium as above, in the presence or absence of 10 µM of the receptor antagonist TLV, or with 0.1% DMSO (controls) for 6 h at 18°C. After incubation, follicles were fixed for immunofluorescence microscopy, frozen in liquid nitrogen and stored at -80°C for further protein extraction, or processed for water uptake assays using radiolabeled water as previously described (12).

### 4.10 ELISA determination of Avp in treated ovarian explants *in vitro*

To determine the Avp levels in ovarian explants incubated *in vitro*, a competitive ELISA was developed using the specific anti-Avp antiserum and the synthetic Avp to create the standard curve (**Supplementary Figure S3**). For this, polystyrene ELISA microtiter plates (F96 Maxisorp Nunc Immuno Plates; Nunc) were coated with 100 µl/well of 50 ng Avp/ml carbonate buffer (50 mM sodium carbonate, pH 9.6) and incubated overnight at 4°C. For the blank and the non-specific binding, two wells were either not coated or coated with 100 µl/well of 50 ng BSA/ml carbonate buffer, respectively. Ovarian explants were homogenized on ice with a glass dounce tissue homogenizer in water containing 0.25% glacial acetic acid in a proportion of 500 µl/100 mg tissue. After incubation of the homogenate at 100°C for 5 min, the samples were centrifuged a 10,000 × *g* for 5 min at 4°C, and the supernatant recovered in a new tube. The standards (0.01-500 ng Avp/ml) and experimental samples were diluted in PBST (PBS plus 0.05% Tween 20) with 0.5% normal goat serum (NGS), containing the Avp antibody diluted at 1:15,000, and were pre-incubated overnight at 4°C. Displacement curves for ovarian samples were obtained by serial dilutions from 1:2 to 1:16 to confirm the parallelism with the standard curves. For the measurement of the experimental samples a 1:4 dilution was applied, and to evaluate the cross-reactivity of the assay with Oxt, the same standard curve as for Avp was created (0.01-500 ng/ml). All standards and samples were processed in duplicate. After coating, the plates were washed 3 times with 200 µl/well of PBST, and blocked with 200 µl/well of PBST buffer containing 2% NGS for 1 h at room temperature. Then, 100 µl of samples and standards preincubated with the antibody were added to each well, and plates were incubated at 4°C for another 24 h. The plates were finally washed 3 times in PBST and each well was incubated with 100 µl/well of EIA grade affinity purified goat anti-rabbit IgG (H + L) HRP conjugated (Bio-rad Laboratories, Inc.) diluted 1:3000 in PBST plus 0.5% NGS for 2 h at room temperature. After washing, enzymatic color development was carried out by the addition of 100 µl/well of tetramethylbenzidine (TMB) peroxidase EIA substrate kit (Bio-rad Laboratories, Inc.) in darkness during 30 min, and the reaction was stopped with 100 µl/well of 1 N sulfuric acid. Absorbance was read at 450 nm with a VICTOR3™ Multilabel Plate Reader (PerkinElmer, Inc.). The amount of Avp was normalized to the wet weight of the ovarian explants. The intra-assay coefficient of variation was 4.64% (calculated by assaying eight replicates of an ovarian sample at 50% of maximum binding on the same plate), whereas the inter-assay coefficient of variation was 8.35%. The sensitivity of the assay for Avp was of 10 pg/ml, while its crossreaction with Oxt was calculated to be 8.43 ± 1.72%.

### 4.11 Immunofluorescence microscopy

Isolated ovarian follicles were processed for immunofluorescence microscopy as previously described (65, 66). Control sections were incubated with antibodies (1:400 dilution) preadsorbed with the corresponding peptides used for immunization (for aquaporin antibodies), or with the Avp or Oxt synthetic peptides. Secondary antibodies were sheep anti-rabbit IgG Cy3 (Merck #C2306) or goat anti-chicken IgY Alexa Fluor 488 (Invitrogen #A11039) at a 1:1,000 dilution. Sections were counterstained with DAPI (1:10,000), and mounted with Fluoromount™ Aqueous Mounting Medium (Merck #F4680). Immunofluorescence was observed and documented with a Zeiss Axio Imager Z1/Apotome fluorescent microscope (Carl Zeiss Corp.). Changes in Aqp1ab1 and Aqp1ab2 in the oocyte plasma membrane after Avp treatment were evaluated using the ImageJ software v.1.52o (https://imagej.nih.gov/ij/) as previously described (12).

### 4.12 Protein extraction and immunoblotting

The total and plasma membrane fractions of *X. laevis* oocytes (*n* = 10), and total membrane fractions of seabream ovarian follicles (*n* = 50), were isolated as described previously (12) and resuspended in Laemmli sample buffer. For immunoblotting, Laemmli-mixed protein samples were denatured at 95°C for 10 min and subjected to 12% sodium dodecyl sulfate polyacrylamide gel electrophoresis (SDS-PAGE) and blotted onto Immun-Blot nitrocellulose 0.2 μm membranes (Bio-Rad Laboratories, Inc.), as described previously (67). Membranes were blocked with 5% nonfat dry milk in TBST (20 mM Tris, 140 mM NaCl, 0.1% Tween, pH 8) for 1 h at room temperature, and subsequently incubated overnight at 4°C with the seabream-specific Aqp1aa, Aqp1ab1 or Aqp1ab2 antisera, or HA antibodies, diluted (1:1,000) in TBST with 5% milk. Bound antibodies were detected with horseradish peroxidase-coupled anti-rabbit (Bio-Rad Laboratories, Inc., #172-1019) or anti-chicken (Thermo Fisher Scientific, #A16054) secondary antibodies diluted 1:5000 as above, and reactive protein bands were revealed using ImmobilonTM Western chemiluminescent HRP substrate (Millipore, #WBKLS).

### 4.13 Co-immunoprecipitation

*X. laevis* oocytes and seabream follicles (*n* = 10) were homogenized in the lysis buffer from the Pierce™ Crosslink Magnetic IP/Co-IP Kit (Pierce Thermo Fisher Scientific, #88805) with a bouncer on ice, and centrifuged at 14,000 × *g* for 1 min. An aliquot (10%) of the protein extract was collected as “input” and mixed with 2 × Laemmli sample buffer, whereas the remaining extract was incubated overnight at 4°C under constant agitation with magnetic beads previously coated with anti-Aqp1ab1 or anti-Aqp1ab2 antibodies. For rabbit primary antibodies, the immunoprecipitation was carried out using the kit indicated above, and final eluted proteins in 50 µl of elution buffer were mixed with 4 × Laemmli sample buffer supplemented with protease and phosphatase inhibitors. For chicken antibodies, the immunoprecipitation was performed using Dynabeads™ M-280 Tosylactivated (Invitrogen Thermo Fisher Scientific, #14203) following the manufacturer’s instructions. The input and immunoprecipitated samples were immunoblotted as indicated above.

### 4.14 Statistical analyses

Comparisons between two independent groups were made by the two-tailed unpaired Student’s *t*-test. The statistical significance among multiple groups was analyzed by one-way ANOVA, followed by the Tukey’s multiple comparison test, or by the non-parametric Kruskal-Wallis test and further Dunn’s test for nonparametric *post hoc* comparisons. Percentages were square root transformed previous analyses. Statistical analyses were carried out using the GraphPad Prism v9.1.2 (226) (GraphPad Software).

## Data availability statement

The original contributions presented in the study are included in the article/**Supplementary Material**. Further inquiries can be directed to the corresponding author.

## Ethics statement

The animal study was reviewed and approved by Procedures relating to the care and use of fish and sample collection were approved by the Ethics Committee of IRTA, following the European Union Council Guidelines (86/609/EU). The procedure for surgical laparotomy of female frogs was approved by the Ethics Committee for Animal and Human Experimentation from UAB and the Catalan Government (Direcció General de Polítiques Ambientals i Medi Natural; Project no. 10985).

## Author contributions

Conceptualization: JC. Investigation: AF, FC and MG. Formal analysis: AF, FC, MG, EK, RF and JC. Supervision: JC and EK. Funding acquisition: JC and RF. Visualization: JC. Writing-original draft preparation: AF. Writing- review & editing: JC, RF and EK. All authors approved the submitted version of the manuscript.
