# Supplementary material for: Neurohypophysial and paracrine vasopressinergic signaling regulates aquaporin trafficking to hydrate marine teleost oocytes
Source: Front Endocrinol (Lausanne). 2023 Aug 11;14:1222724. doi: 10.3389/fendo.2023.1222724 (PMC10454913; doi:10.3389/fendo.2023.1222724)
Supplement: Supplementary file 1 [file DataSheet_1.pdf]

Supplementary Table S1. Primers used to amplify the seabream mRNAs encoding Avp, Oxt and corresponding receptors

Transcript	GenBank accession no.	Direction	Primer sequence (5’ to 3’)	Purpose
pro-avp	FR851924	Forward	TGAACATCATCCCTGTTCCA	RT-PCR
		Reverse	ACTTTTGCAAAGCCGTCATC	
		Forward	TGAACATCATCCCTGTTCCA	ISH probe
		Reverse	TTTTGGCAGCGATTCAGTCT	
pro-oxt	FR851925	Forward	ATGACCAAAGCAGCCAATTC	RT-PCR
		Reverse	GGCATCAGATGATGACAAACA	
		Forward	CTGCTGACGCAATGGGACTA	ISH probe
		Reverse	TCAAAACTTTATTTCAACATGCAG	
avpr1aa	KC195974	Forward	ACTATGCGCTTGTCCTGAGC	Cloning of Full-length cDNA and RT-PCR
		Reverse	TGTGTACCTTGATGCCAGACA	
		Forward	TCACCTTCTCCAGGATTTCG	
		Forward	AGAAGGAGGACTCAGACAGCA	ISH probe
		Reverse	TGTGTACCTTGATGCCAGACA	
avpr2aa	KC960488	Forward	AAAGGACACGCGTGAGAAAG	Cloning of Full-length cDNA and RT-PCR
		Reverse	CCGTGCATGTCTTTTCAAAC	
		Forward	CCAAGGAGTAGCCTTCACCA	
		Forward	ACGGCTTCAAGAAGGAGGAC	ISH probe
		Reverse	GGAGAGGGGGTGCGTAGT	
oxtrb	KC195973	Forward	GACCCGGACTCTTGTGTTGT	Cloning of Full-length cDNA and RT-PCR
		Reverse	GGGATTGCCAGGTTACTCAA	
		Forward	ATCCTGGCTCTGACTGCAAG	
		Forward	GTACCTGACAGCCTCCACCT	ISH probe
		Reverse	TTGGGATTGCCAGGTTACTC	
rps18	AY587263	Forward	ACTAAGAACGGCCATGCACCACCAC	RT-PCR
		Reverse	GAATTGACGGAAGGGCACCACC	
