# Supplementary material for: Neurohypophysial and paracrine vasopressinergic signaling regulates aquaporin trafficking to hydrate marine teleost oocytes
Source: Front Endocrinol (Lausanne). 2023 Aug 11;14:1222724. doi: 10.3389/fendo.2023.1222724 (PMC10454913; doi:10.3389/fendo.2023.1222724)
Supplement: Supplementary file 2 [file DataSheet_2.pdf]

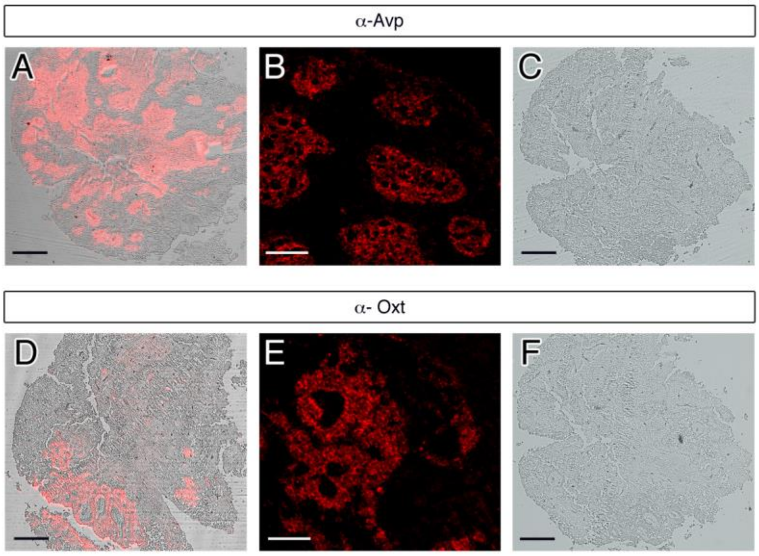

Figure S1. Immunolocalization of Avp and Oxt in the seabream pituitary gland.

(A-E) Immunostaining of Avp (A-C) and Oxt (D-F) in paraffin sections of the posterior pituitary (neurohypophysis). In A and C and D and F, the epifluorescence image (red color) is merged with the brightfield image. **(C-F)** Control sections incubated with preabsorbed antisera. Scale bars, 200 µm (A, D, C and F); 50 µm (B and E).

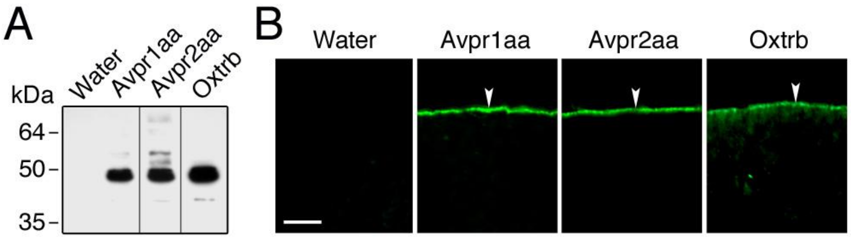

Figure S2. Expression of seabream Avp and Oxt receptors in *X. laevis* oocytes.

(A) Immunoblots of total membranes of oocytes injected with water or expressing HA-tagged Avpr1aa, Avpr2aa or Oxtrb. Molecular mass markers (kilodaltons) are on the left. **(B)** Immunofluoresence microscopy of water-injected and Avpr1aa-, Avpr2aa- or Oxtrb-expressing oocytes. The arrowhead points the oocyte plasma membrane. Scale bar, 50 µm.

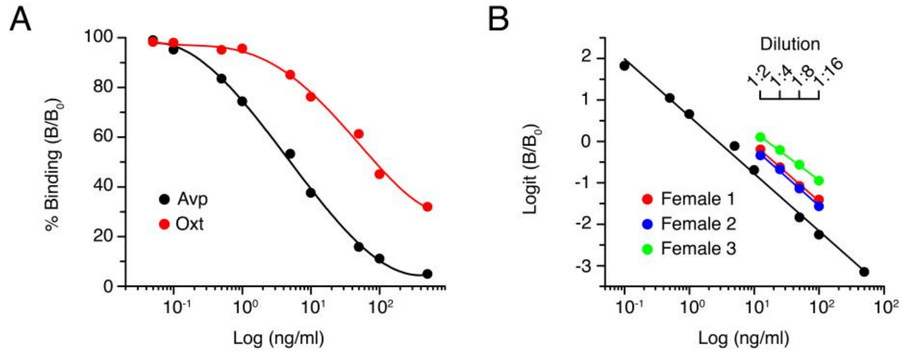

Figure S3. Development of an ELISA for Avp.

(A) Competitive binding curves for arginine vasopressin (Avp) standard, and cross-reactivity with serial dilutions of oxytocin (Oxt), using the anti-Avp antiserum. Data are the mean of 3 replicates. The optimal concentration for coating the wells with Avp was 50 ng/ml using a 1:15000 dilution of the antibody, whereas the Avp used to generate the standard curves ranged from 10 pg to 500 ng/ml. The sensitivity of the assay was 10 pg/ml (B/B0 > 95%) with the optical density decreasing as a linear function of Avp concentration (r^2 =0.9945). The intra- and inter-assay coefficient of variation was 4.64% and 8.35%, respectively. The evaluation of the cross-reactivity of the assay with Oxt showed that the displacement of the B/B0 dilution curve obtained with Avp was only seen when using high doses of Oxt (>10 ng/ml), the cross-reaction being 8.4%. **(B)** Parallelism between the standard curve of ELISA using Avp and displacement curves obtained with serial dilutions of the ovary from three different females. Each point is the mean of two replicates. The slope obtained for Avp (slope ± SEM) was of −0.588 ± 0.025 , and not significantly different from that obtained for ovarian samples from 3 different females (-0.566 ± 0.029).

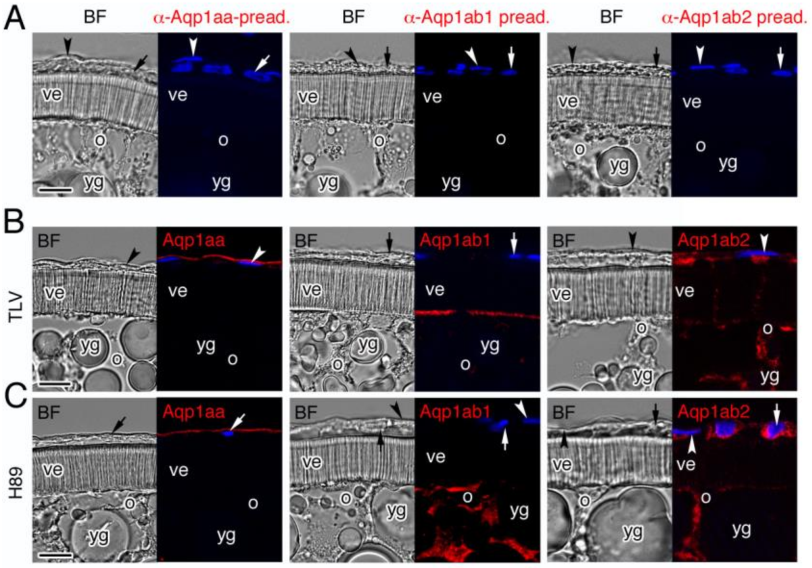

Figure S4. Immunostaining of the TSA1C channels in the seabream ovarian follicle.

(A) Control paraffin sections incubated with preabsorbed antisera specific for Aqp1aa, Aqp1ab1 and Aqp1ab2. **(B-C)** Representative immunostaining of Aqp1aa, Aqp1ab1 and Aqp1ab2 (red color) in follicles exposed only to 10 µM of the Avpr2aa antagonist tolvaptan (TLV) or the PKA inhibitor H89. In all panels, brightfield (BF) and epifluprescence images are shown on the left and right, respectively, and nuclei were counterstained with DAPI (blue). The theca and granulosa cells are indicated by arrows and arrowheads, respectively. ve, vitelline envelope; o, oocyte; yg, yolk globules. Scale bars, 10 µm.

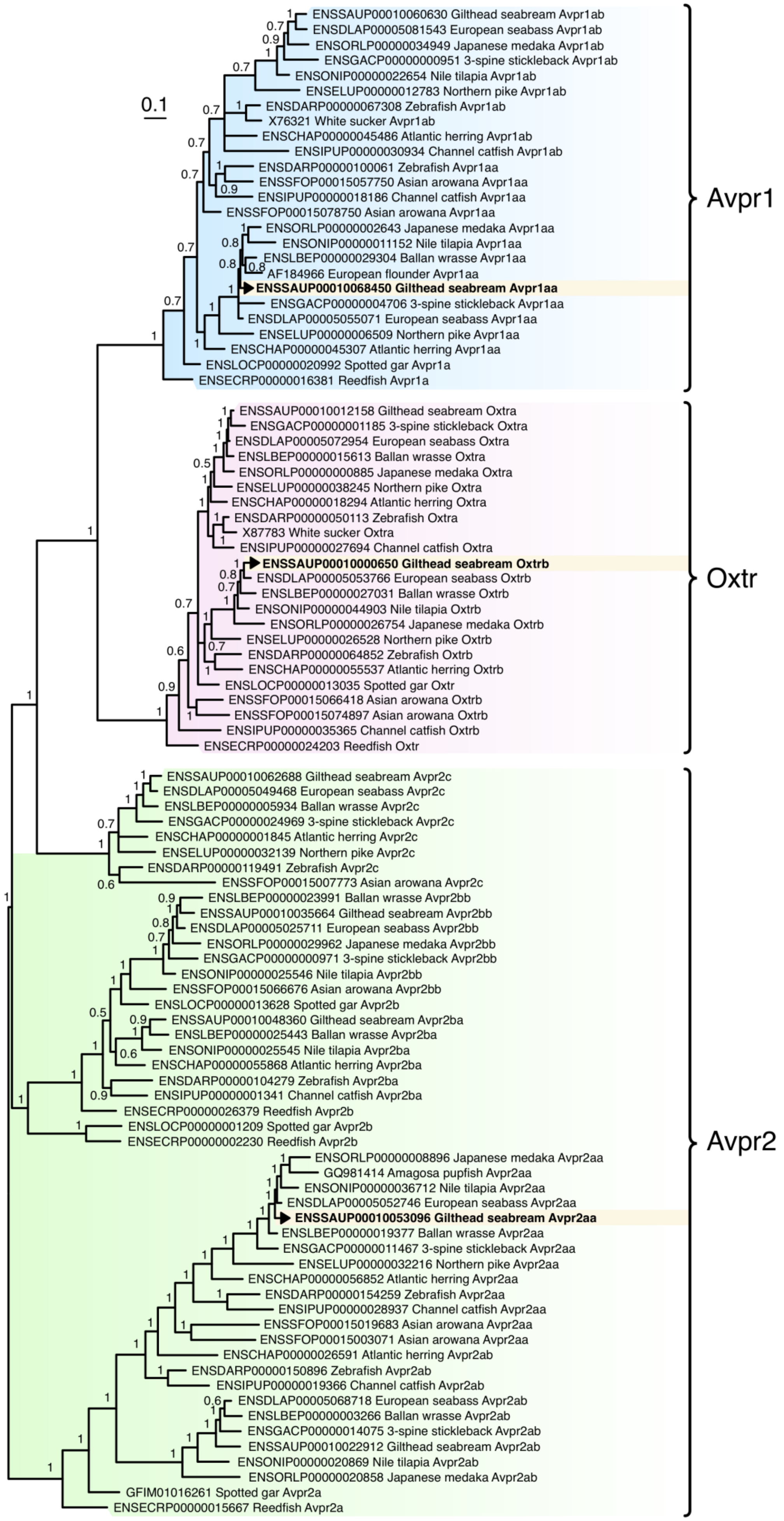
0.1
ENSSAUP00010060630 Gilthead seabream Avpr1ab
ENSDLAP00005081543 European seabass Avpr1ab
ENSORLP00000034949 Japanese medaka Avpr1ab
ENSGACP00000000951 3-spine stickleback Avpr1ab
ENSONIP00000022654 Nile tilapia Avpr1ab
ENSELUP00000012783 Northern pike Avpr1ab
ENSDARP00000067308 Zebrafish Avpr1ab
X76321 White sucker Avpr1ab
ENSCHAP00000045486 Atlantic herring Avpr1ab
ENSIPUP00000030934 Channel catfish Avpr1ab
ENSDARP00000100061 Zebrafish Avpr1aa
ENSSFOP00015057750 Asian arowana Avpr1aa
ENSIPUP00000018186 Channel catfish Avpr1aa
ENSSFOP00015078750 Asian arowana Avpr1aa
ENSORLP00000002643 Japanese medaka Avpr1aa
ENSONIP00000011152 Nile tilapia Avpr1aa
ENSLBEP00000029304 Ballan wrasse Avpr1aa
AF184966 European flounder Avpr1aa
ENSSAUP00010068450 Gilthead seabream Avpr1aa
ENSGACP00000004706 3-spine stickleback Avpr1aa
ENSDLAP00005055071 European seabass Avpr1aa
ENSELUP00000006509 Northern pike Avpr1aa
ENSCHAP00000045307 Atlantic herring Avpr1aa
ENSLOCP00000020992 Spotted gar Avpr1a
ENSECRP00000016381 Reedfish Avpr1a
Avpr1
ENSSAUP00010012158 Gilthead seabream Oxtra
ENSGACP00000001185 3-spine stickleback Oxtra
ENSDLAP00005072954 European seabass Oxtra
ENSLBEP00000015613 Ballan wrasse Oxtra
ENSORLP00000000885 Japanese medaka Oxtra
ENSELUP00000038245 Northern pike Oxtra
ENSCHAP00000018294 Atlantic herring Oxtra
ENSDARP00000050113 Zebrafish Oxtra
X87783 White sucker Oxtra
ENSIPUP00000027694 Channel catfish Oxtra
ENSSAUP00010000650 Gilthead seabream Oxtrb
ENSDLAP00005053766 European seabass Oxtrb
ENSLBEP00000027031 Ballan wrasse Oxtrb
ENSONIP00000044903 Nile tilapia Oxtrb
ENSORLP00000026754 Japanese medaka Oxtrb
ENSELUP00000026528 Northern pike Oxtrb
ENSDARP00000064852 Zebrafish Oxtrb
ENSCHAP00000055537 Atlantic herring Oxtrb
ENSLOCP00000013035 Spotted gar Oxtr
ENSSFOP00015066418 Asian arowana Oxtrb
ENSSFOP00015074897 Asian arowana Oxtrb
ENSIPUP00000035365 Channel catfish Oxtrb
ENSECRP00000024203 Reedfish Oxtr
Oxtr
ENSSAUP00010062688 Gilthead seabream Avpr2c
ENSDLAP00005049468 European seabass Avpr2c
ENSLBEP00000005934 Ballan wrasse Avpr2c
ENSGACP00000024969 3-spine stickleback Avpr2c
ENSCHAP00000001845 Atlantic herring Avpr2c
ENSELUP00000032139 Northern pike Avpr2c
ENSDARP00000119491 Zebrafish Avpr2c
ENSSFOP00015007773 Asian arowana Avpr2c
ENSLBEP00000023991 Ballan wrasse Avpr2bb
ENSSAUP00010035664 Gilthead seabream Avpr2bb
ENSDLAP00005025711 European seabass Avpr2bb
ENSORLP00000029962 Japanese medaka Avpr2bb
ENSGACP00000000971 3-spine stickleback Avpr2bb
ENSONIP00000025546 Nile tilapia Avpr2bb
ENSSFOP00015066676 Asian arowana Avpr2bb
ENSLOCP00000013628 Spotted gar Avpr2b
ENSSAUP00010048360 Gilthead seabream Avpr2ba
ENSLBEP00000025443 Ballan wrasse Avpr2ba
ENSONIP00000025545 Nile tilapia Avpr2ba
ENSCHAP00000055868 Atlantic herring Avpr2ba
ENSDARP00000104279 Zebrafish Avpr2ba
ENSIPUP00000001341 Channel catfish Avpr2ba
ENSECRP00000026379 Reedfish Avpr2b
ENSLOCP00000001209 Spotted gar Avpr2b
ENSECRP00000002230 Reedfish Avpr2b
ENSORLP00000008896 Japanese medaka Avpr2aa
GQ981414 Amagosa pupfish Avpr2aa
ENSONIP00000036712 Nile tilapia Avpr2aa
ENSDLAP00005052746 European seabass Avpr2aa
ENSSAUP00010053096 Gilthead seabream Avpr2aa
ENSLBEP00000019377 Ballan wrasse Avpr2aa
ENSGACP00000011467 3-spine stickleback Avpr2aa
ENSELUP00000032216 Northern pike Avpr2aa
ENSCHAP00000056852 Atlantic herring Avpr2aa
ENSDARP00000154259 Zebrafish Avpr2aa
ENSIPUP00000028937 Channel catfish Avpr2aa
ENSSFOP00015019683 Asian arowana Avpr2aa
ENSSFOP00015003071 Asian arowana Avpr2aa
ENSCHAP00000026591 Atlantic herring Avpr2ab
ENSDARP00000150896 Zebrafish Avpr2ab
ENSIPUP00000019366 Channel catfish Avpr2ab
ENSDLAP00005068718 European seabass Avpr2ab
ENSLBEP00000003266 Ballan wrasse Avpr2ab
ENSGACP00000014075 3-spine stickleback Avpr2ab
ENSSAUP00010022912 Gilthead seabream Avpr2ab
ENSONIP00000020869 Nile tilapia Avpr2ab
ENSORLP00000020858 Japanese medaka Avpr2ab
GFIM01016261 Spotted gar Avpr2a
ENSECRP00000015667 Reedfish Avpr2a
Avpr2

Figure S5: Molecular phylogeny of piscine vasopressinergic and oxytocinergic receptors.

Bayesian majority rule midpoint rooted consensus tree inferred from 2 million MCMC generations (aamodel = mixed4; burnin = 25%) of a ClustalX amino acid alignment of 97 receptors downloaded from Ensembl (v109). Bayesian posterior probabilities are shown at each node with the scale bar indicating the expected rate of amino acid substitution per site. The seabream paralogs used for functional experiments in the present study are highlighted (orange) with arrows in bold. The tree topology matches that computed by maximum likelihood (Daza et al., 2022) with the exception that the zebrafish Avpr2ba and Avpr2bb receptors are named in accordance with the Ensembl (v109) nomenclature.

Daza DO, Bergqvist CA, Larhammar D (2022) The evolution of oxytocin and vasotocin receptor genes in jawed vertebrates: A clear case for gene duplications through ancestral whole-genome duplications. Front Endocrinol 12: 792644.
